# Oxygen, angiogenesis, cancer and immune interplay in breast tumour microenvironment: a computational investigation

**DOI:** 10.1098/rsos.240718

**Published:** 2024-12-11

**Authors:** Navid Mohammad Mirzaei, Panayotis G. Kevrekidis, Leili Shahriyari

**Affiliations:** ^1^ Department of Epidemiology, Mailman School of Public Health, Columbia University, New York 10032, USA; ^2^ Department of Mathematics and Statistics, University of Massachusetts Amherst, Amherst, MA 01003-4515, USA

**Keywords:** breast cancer, tumour microenvironment, angiogenesis, mathematical modelling

## Abstract

Breast cancer is a challenging global health problem among women. This study investigates the intricate breast tumour microenvironment (TME) dynamics utilizing data from mammary-specific polyomavirus middle T antigen overexpression mouse models (MMTV-PyMT). It incorporates endothelial cells (ECs), oxygen and vascular endothelial growth factors (VEGF) to examine the interplay of angiogenesis, hypoxia, VEGF and immune cells in cancer progression. We introduce an approach to impute immune cell fractions within the TME using single-cell RNA-sequencing (scRNA-seq) data from MMTV-PyMT mice. We quantify our analysis by estimating cell counts using cell size data and laboratory findings from existing literature. We perform parameter estimation via a Hybrid Genetic Algorithm (HGA). Our simulations reveal various TME behaviours, emphasizing the critical role of adipocytes, angiogenesis, hypoxia and oxygen transport in driving immune responses and cancer progression. Global sensitivity analyses highlight potential therapeutic intervention points, such as VEGFs’ role in EC growth and oxygen transportation and severe hypoxia’s effect on cancer and the total number of cells. The VEGF-mediated production rate of ECs shows an essential time-dependent impact, highlighting the importance of early intervention in slowing cancer progression. These findings align with clinical observations demonstrating the VEGF inhibitors’ efficacy and suggest a timely intervention for better outcomes.

## 1. Introduction

Breast cancer is the most prevalent cancer among women globally [1], with 2.3 million new cases and 685 000 deaths reported in 2022 [2]. The estimated cases for the United States in 2023 are 298 000, with 43 000 deaths [3]. Surgical removal followed by radiotherapy is a standard treatment for non-metastatic breast cancer, varying based on subtypes such as HR+, ERBB2+ and triple-negative [4]. For example, endocrine therapy is prescribed for HR+ subtypes and trastuzumab-based ERBB2-directed antibody therapy in addition to chemotherapy for ERBB2+ subtypes and chemotherapy alone for triple-negative subtypes. Understanding the complex cellular and molecular interplays of the tumour microenvironment (TME) is crucial in achieving more effective treatment strategies [5].

The TME, an ecosystem including tumour cells, immune cells, fibroblasts, blood vessels, cytokines and the extracellular matrix, is an active promoter of cancer progression [6]. New technologies are developed to study the intricacies within the TME, such as spatial transcriptomics and multiplexed proteomics [7]. Longitudinal *in vivo* investigations of the TME are costly and straining, so an alternative is using genetically engineered mouse models [8–10].

The mammary-specific polyomavirus middle T antigen overexpression mouse model (MMTV-PyMT) is one of the most popular mouse models since its discovery in 1992 [11–14], developing spontaneous mammary tumours similar to human breast cancers [15]. The histopathology and breast cancer biomarkers expression in the MMTV-PyMT tumours is in line with late-stage human breast cancers [16].

Despite mouse models’ convenience, making predictions about cells and cytokine dynamics in the TME is still challenging. However, mathematical models can help scientists make valuable estimations, predictions or hypotheses based on limited data. Some mathematical cancer models focus on a certain cell type mutation [17,18], some investigate a set of interactions in the TME [19–21], others aim to discover optimal treatment dosage [22–24] and some describe it as an epidemiological problem and search for the contributing risk factors [25,26].

Among the crucial mechanisms in TME benefiting from mathematical modelling are those leading to vessel formation (angiogenesis) and oxygen delivery. Chaplain has introduced a generic tumour angiogenesis model based on the ECs, angiogenic cytokine and fibronectin interactions [27]; see also the earlier impactful work of [28]. Harrington *et al*. designed a mathematical model to simulate vessel formation in the cornea in the presence of promoter and inhibitor cytokines [29], following the earlier work of [30]. Both these and other related (see, e.g. the review of [31]) studies focus on the vessels’ spatial distribution and obtain valuable results agreeing with relevant biological observations. Nevertheless, considerable space exists for further developments, notably the tumour angiogenic factors (so-called TAFs) and TME interactions.

Angiogenesis, a critical process in tumour progression, plays a central role in breast cancer development. Tumours require a constant blood supply to sustain their growth, and angiogenesis ensures new blood vessel formation. The solid TME often experiences hypoxia, a condition of insufficient oxygen levels. Hypoxia triggers the VEGF upregulation, a key pro-angiogenic factor. VEGF promotes EC proliferation and migration, leading to new blood vessel formation. The interactions in the TME can play a significant role in promoting or inhibiting angiogenesis. The intricate interplays between the TME, angiogenesis, hypoxia and VEGF underscore the significance of targeting these pathways for therapeutic interventions. Numerous studies have investigated these mechanisms, with seminal works including Folkman [32] and Ferrara *et al*. [33].

In this paper, we propose an ODE model describing the interactions in the TME, an extension of a previous study [34]. The extension concerns angiogenesis and oxygen delivery mechanisms, i.e. we include three major players: ECs, oxygen and VEGF. While spatial interactions among these elements are not considered for simplicity, we acknowledge their significance as a potential future research direction. Here, we present a methodology to prepare scRNA-seq data for an ODE system describing TME interactions. Using a Hybrid Genetic Algorithm (HGA), we estimate parameters and discuss simulation outcomes, augmented by sensitivity analyses to uncover influential mechanisms on the system, cancer cells and total immune cells. Our findings highlight the significance of pathways related to angiogenesis, oxygenation and hypoxia.

### 1.1. The model

**Figure 1.**
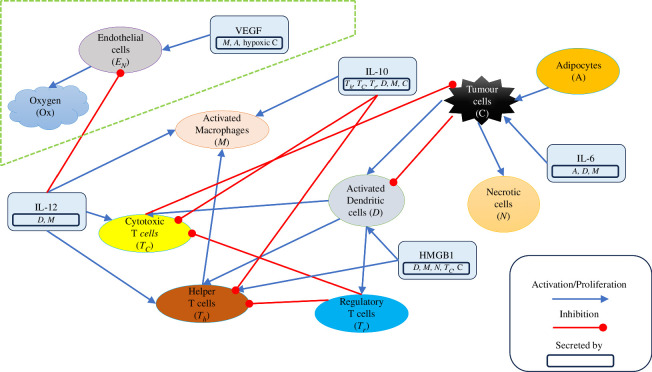
Interaction network. The portion inside the dashed shape is an addition to the network used by Mohammad Mirzaei *et al*. [34,35]. Despite the importance of oxygen in the network, no arrows are coming out of it. This is because lack of oxygen (hypoxia) is the key factor, not oxygen itself.

The model in this paper extends the framework established by Mohammad Mirzaei *et al*. [34]. In our adaptation, we have incorporated additional state variables: VEGF, ECs and oxygen. These new elements are added to investigate hypoxia and angiogenesis roles in tumour progression. For a detailed representation of the interaction network, see figure 1. The newly incorporated interactions are distinctly marked for easy identification. Table 1 shows the model variables with their corresponding equations.

**Table 1.** Model variables with their equations.

variable	name	equation for their rate of changes
$E_{N}$	ECs	$\begin{array}{l} \frac{d[\bar{E_{N}}]}{dt}={\bar{\lambda }}_{VE_{N}}[\bar{V}](1-\frac{\left[\bar{E_{N}}\right]}{{\bar{E_{N}}}_{0}})[\bar{E_{N}}]-{\bar{\delta }}_{E_{N}IL_{12}}[\bar{IL_{12}}][\bar{E_{N}}]-(\delta_{E_{N}}+{\bar{\delta }}_{E_{N}O_{x}}f_{2}([\bar{O_{x}}]))[\bar{E_{N}}] \end{array}$
V	VEGF	$\begin{array}{l} \frac{d[\bar{V}]}{dt}={\bar{\lambda }}_{VA}[\bar{A}]+{\bar{\lambda }}_{VM}[\bar{M}]+{\bar{\lambda }}_{VCO_{x}}[\bar{C}]f_{1}(O_{x})+{\bar{\lambda }}_{VO_{x}}f_{1}([\bar{O_{x}}])-\delta_{V}[\bar{V}] \end{array}$
$O_{x}$	oxygen	$\begin{array}{l} \frac{d[\bar{O_{x}}]}{d\tau }={\bar{\lambda }}_{O_{x}E_{N}}([\bar{E_{N}}])-{\bar{\delta }}_{1}\left(\left[\bar{T_{N}}]+[\bar{T_{h}}]+[\bar{T_{c}}]+[\bar{T_{r}}\right]\right)[\bar{O_{x}}]-{\bar{\delta }}_{2}\left(\left[\bar{D_{N}}]+[\bar{D}\right]\right)[\bar{O_{x}}]-{\bar{\delta }}_{3}\left(\left[\bar{M_{N}}]+[\bar{M}\right]\right)[\bar{O_{x}}]-{\bar{\delta }}_{4}[\bar{C}][\bar{O_{x}}]-{\bar{\delta }}_{5}[\bar{A}][\bar{O_{x}}]-{\bar{\delta }}_{6}[\bar{E_{N}}][\bar{O_{x}}]-\delta_{O_{x}}[\bar{O_{x}}] \end{array}$
$T_{N}$	naive T-cells	$\begin{array}{l} \frac{d[\bar{T_{N}}]}{dt}={\bar{A}}_{T_{N}}-\left({\bar{\lambda }}_{T_{h}H}[\bar{H}]+{\bar{\lambda }}_{T_{h}D}[\bar{D}]+{\bar{\lambda }}_{T_{h}IL_{12}}[\bar{IL_{12}}]\right)[\bar{T_{N}}]-\left({\bar{\lambda }}_{T_{c}D}[\bar{D}]+{\bar{\lambda }}_{T_{c}IL_{12}}[\bar{IL_{12}}]\right)[\bar{T_{N}}]-\left({\bar{\lambda }}_{T_{r}D}[\bar{D}]+{\bar{\lambda }}_{T_{r}Ox}f_{1}([\bar{Ox}])\right)[\bar{T_{N}}]-\left(\delta_{T_{N}}+{\bar{\delta }}_{T_{N}Ox}f_{2}([\bar{Ox}])\right)[\bar{T_{N}}] \end{array}$
$T_{h}$	helper T-cells	$\begin{array}{l} \frac{d[\bar{T_{h}}]}{dt}=\left({\bar{\lambda }}_{T_{h}H}[\bar{H}]+{\bar{\lambda }}_{T_{h}D}[\bar{D}]+{\bar{\lambda }}_{T_{h}IL_{12}}[\bar{IL_{12}}]\right)[\bar{T_{N}}]-\left({\bar{\delta }}_{T_{h}T_{r}}[\bar{T_{r}}]+{\bar{\delta }}_{T_{h}IL_{10}}[\bar{IL_{10}}]\right)[\bar{T_{h}}]-\left(\delta_{T_{h}}+{\bar{\delta }}_{T_{h}O_{x}}f_{2}([\bar{O_{x}}])\right)[\bar{T_{h}}] \end{array}$
$T_{C}$	cytotoxic cells	$\begin{array}{l} \frac{d[\bar{T_{c}}]}{dt}=\left({\bar{\lambda }}_{T_{c}D}[\bar{D}]+{\bar{\lambda }}_{T_{c}IL_{12}}[\bar{IL_{12}}]\right)[\bar{T_{N}}]-\left({\bar{\delta }}_{T_{c}T_{r}}[\bar{T_{r}}]+{\bar{\delta }}_{T_{c}IL_{10}}[\bar{IL_{10}}]\right)[\bar{T_{c}}]-\left(\delta_{T_{c}}+{\bar{\delta }}_{T_{c}O_{x}}f_{2}([\bar{O_{x}}])\right)[\bar{T_{c}}] \end{array}$
$T_{r}$	regulatory T-cells	$\begin{array}{l} \frac{d[\bar{T_{r}}]}{dt}=\left({\bar{\lambda }}_{T_{r}D}[\bar{D}]+{\bar{\lambda }}_{T_{r}O_{x}}\;f_{1}([\bar{O_{x}}])\right)\;[\bar{T_{N}}]-\left(\delta_{T_{r}}+{\bar{\delta }}_{T_{r}O_{x}}f_{2}([\bar{O_{x}}])\right)[\bar{T_{r}}] \end{array}$
$D_{N}$	naive DCs	$\begin{array}{l} \frac{d[\bar{D_{N}}]}{dt}={\bar{A}}_{D_{N}}-\left({\bar{\lambda }}_{DC}[\bar{C}]+{\bar{\lambda }}_{DH}[\bar{H}]+{\bar{\lambda }}_{DO_{x}}f_{1}([\bar{O_{x}}])\right)[\bar{D_{N}}]-\left(\delta_{D_{N}}+{\bar{\delta }}_{D_{N}O_{x}}f_{2}([\bar{O_{x}}])\right)[\bar{D_{N}}] \end{array}$
D	activated DCs	$\begin{array}{l} \frac{d[\bar{D}]}{dt}=\left({\bar{\lambda }}_{DC}[\bar{C}]+{\bar{\lambda }}_{DH}[\bar{H}]+{\bar{\lambda }}_{DO_{x}}f_{1}([\bar{O_{x}}])\right)[\bar{D_{N}}]-\left({\bar{\delta }}_{DC}[\bar{C}]+\delta_{D}+{\bar{\delta }}_{D_{N}O_{x}}f_{2}([\bar{O_{x}}])\right)[\bar{D}] \end{array}$
$M_{N}$	naive macrophages	$\begin{array}{l} \frac{d[\bar{M_{N}}]}{dt}={\bar{A}}_{M_{N}}-({\bar{\lambda }}_{MIL_{10}}[\bar{IL_{10}}]+{\bar{\lambda }}_{MIL_{12}}[\bar{IL_{12}}]+{\bar{\lambda }}_{MT_{h}}[\bar{T_{h}}]+\delta_{M_{N}})[\bar{M_{N}}] \end{array}$
M	macrophages	$\begin{array}{l} \frac{d[\bar{M}]}{dt}=\left({\bar{\lambda }}_{MIL_{10}}[\bar{IL_{10}}]+{\bar{\lambda }}_{MIL_{12}}[\bar{IL_{12}}]+{\bar{\lambda }}_{MT_{h}}[\bar{T_{h}}]\right)[\bar{M_{N}}]-\delta_{M}[\bar{M}] \end{array}$
C	cancer cells	$\begin{array}{l} \frac{d[\bar{C}]}{dt}=\left(\lambda_{C}+{\bar{\lambda }}_{CIL_{6}}[\bar{IL_{6}}]+{\bar{\lambda }}_{CA}[\bar{A}]\right)\left(1-\frac{\left[\bar{C}\right]}{\bar{C_{0}}}\right)[\bar{C}]-\left({\bar{\delta }}_{CT_{c}}[\bar{T_{c}}]+{\bar{\delta }}_{CT_{c}O_{x}}f_{1}([\bar{O_{x}}])[\bar{T_{c}}]\right)[\bar{C}]-\left({\bar{\delta }}_{CO_{x}}f_{2}([\bar{O_{x}}])+\delta_{C}\right)[\bar{C}] \end{array}$
N	necrotic cells	$\begin{array}{l} \frac{d[\bar{N}]}{dt}={\bar{\alpha }}_{NC}\left({\bar{\delta }}_{CT_{c}}[\bar{T_{c}}]+{\bar{\delta }}_{CT_{c}O_{x}}f_{1}([\bar{O_{x}}])[\bar{T_{c}}]+{\bar{\delta }}_{CO_{x}}f_{2}([\bar{O_{x}}])+\delta_{C}\right)[\bar{C}]-\delta_{N}[\bar{N}] \end{array}$
A	adipocytes	$\begin{array}{l} \frac{d[\bar{A}]}{dt}=\lambda_{A}\left(1-\frac{\left[\bar{A}\right]}{\bar{A_{0}}}\right)[\bar{A}]-\delta_{A}[\bar{A}] \end{array}$
H	HMGB1	$\begin{array}{l} \frac{d[\bar{H}]}{dt}={\bar{\lambda }}_{HD}[\bar{D}]+{\bar{\lambda }}_{HN}[\bar{N}]+{\bar{\lambda }}_{HM}[\bar{M}]+{\bar{\lambda }}_{HT_{c}}[\bar{T_{c}}]+{\bar{\lambda }}_{HC}[\bar{C}]-\delta_{H}[\bar{H}] \end{array}$
$I\,L_{12}$	IL-12	$\begin{array}{l} \frac{d[\bar{IL_{12}}]}{dt}={\bar{\lambda }}_{IL_{12}M}[\bar{M}]+{\bar{\lambda }}_{IL_{12}D}[\bar{D}]-\delta_{IL_{12}}[\bar{IL_{12}}] \end{array}$
$IL_{10}$	IL-10	$\begin{array}{l} \frac{d[\bar{IL_{10}}]}{dt}={\bar{\lambda }}_{IL_{10}M}[\bar{M}]+{\bar{\lambda }}_{IL_{10}D}[\bar{D}]+{\bar{\lambda }}_{IL_{10}T_{r}}[\bar{T_{r}}]+{\bar{\lambda }}_{IL_{10}T_{h}}[\bar{T_{h}}]+{\bar{\lambda }}_{IL_{10}T_{c}}[\bar{T_{c}}]+{\bar{\lambda }}_{IL_{10}C}[\bar{C}]+{\bar{\lambda }}_{IL_{10}O_{x}}f_{1}([\bar{O_{x}}])-\delta_{IL_{10}}[\bar{IL_{10}}] \end{array}$
$IL_{6}$	IL-6	$\begin{array}{l} \frac{d[\bar{IL_{6}}]}{dt}={\bar{\lambda }}_{IL_{6}A}[\bar{A}]+{\bar{\lambda }}_{IL_{6}M}[\bar{M}]+{\bar{\lambda }}_{IL_{6}MO_{x}}[\bar{M}]f_{1}([\bar{O_{x}}])+{\bar{\lambda }}_{IL_{6}D}[\bar{D}]+{\bar{\lambda }}_{IL_{6}O_{x}}f_{1}([\bar{O_{x}}])-\delta_{IL_{6}}[\bar{IL_{6}}] \end{array}$

The naming conventions for our parameters are as follows:


$$\begin{array}{rl} \lambda_{XY} & \;\text{The rate molecule/cell Y promotes the production or proliferation of molecule/cell X}\\ \delta_{XY} & \;\text{The rate molecule/cell Y inhibits the }\text{production or proliferation of molecule/cell X}\\ \delta_{XYOx} & \;\text{The rate molecule/cell Y inhibits the }\text{production of molecule/cell X proportional to}\\ & \;\text{the hypoxia function value}\\ \delta_{X} & \;\text{Natural decay/death rate of molecule/cell X}\\ A_{X} & \;\text{Innate production rate of the cell X}\\ \delta_{i} & \;\text{For }i=1,\cdots ,6.\text{ Oxygen consumption rate by }\text{the six major cell types in the model} \end{array}$$

In this paper, parameters with overhead bars are in non-dimensionalized form. Mathematically, we consider production processes, both for a single species or stimulated by the presence of a second species. Additionally, we account for degradation processes, either occurring naturally for a single species or stimulated by the presence of or interaction with another species.

**Figure 2.**
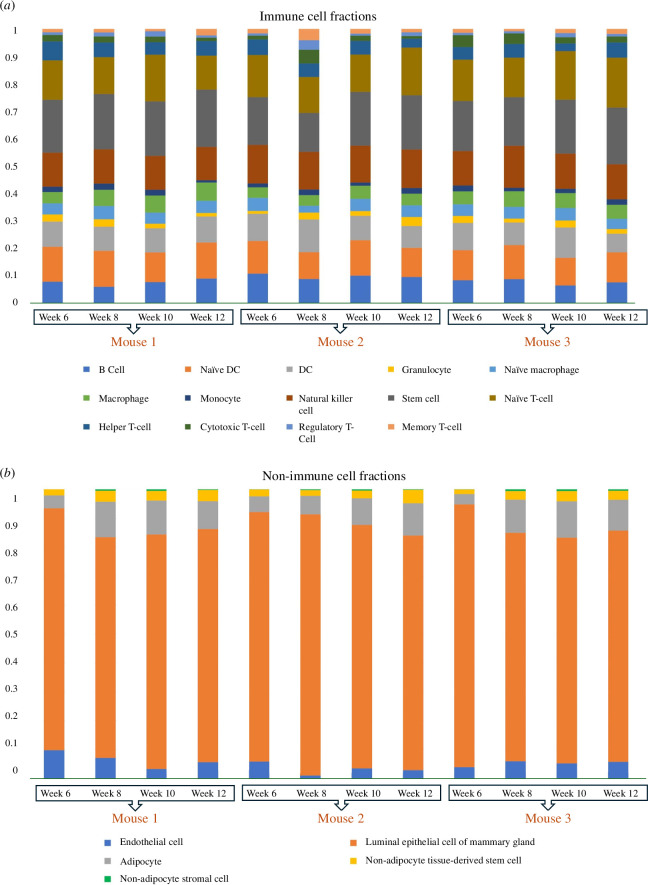
Cell fractions for (*a*) immune cells and (*b*) non-immune cells. The proportions are calculated for three mice at week 6 (hyperplasia), week 8 (adenoma/MIN), week 10 (early carcinoma) and week 12 (late carcinoma).

We used RNA-sequencing data from three MMTV-PyMT mice [36]. The proportions of various immune cells were estimated using the digital cytometry technique CIBERSORTx [37], with the signature matrix extracted from the Immunological Genome Project [38]. We created a unique signature matrix for non-immune cell populations using single-cell RNA sequencing data from the Tabula Muris database [39]. Figure 2 shows imputed fractions for immune and non-immune cells. Additionally, we applied a laboratory protocol tailored for MMTV-PyMT mice to quantify macrophages and cancer cells relative to tumour size. This process approximated the total cell count, informed by imputed cell fractions, and allowed for the direct extraction of cytokine levels from the RNA-sequencing data.

We employed HGA for parameter estimation in our model, followed by a global sensitivity analysis to determine parameters significantly affecting overall system dynamics, cancer and total immune cell counts. Perturbing these sensitive parameters allows us to assess and quantify their impact on model outcomes, as detailed in §2.

## 2. Methods

### 2.1. Model variables and their interactions

We use the mass action law to create the ODE system for the interactions among variables as discussed in [40].

#### 2.1.1. Hypoxia

Hypoxic effects take hours to impact the system [41], while the simulation time for our problem and the associated data is in the order of weeks. Therefore, we assume low oxygen levels entail hypoxic effects on cells for each computational time step corresponding to one day. Usually, laboratory cell cultures are in a hyperoxic condition with about 20.9% oxygenation [42]. However, the *in vivo* normoxic oxygen level in breast tissue is about 8.5%, and the hypoxic level is below 1% [43]. Although different T-cell types have different hypoxic responses at different oxygen levels, most of these hypoxic activities occur in the range 0–1% [44]. In particular, at 0.1–1%, there is not enough oxygen in the environment to disintegrate HIF-1α, so the latter will accumulate and trigger some hypoxic functions and below 0.1% most cell cycles yield, causing hypoxic death [44]. For simplicity, if a particular cell or cytokine is promoted or inhibited via HIF pathways, which are not included in our model, we use a rate independent of other state variables and only dependent on oxygen levels. Some hypoxia outcomes through HIF pathways included in our model are upregulating the VEGF, IL-6 and IL-10 production [45], promoting the maturation of the dendritic cells [46,47], increasing the regulatory T-cells differentiation [48,49] and increasing the T-cells cytotoxicity [50,51]. These outcomes indirectly lead to reduced cytotoxic and helper T-cell differentiation. Also, hypoxia promotes the macrophages’ differentiation into a pro-tumour subtype [52]. However, since we do not include the macrophage subtypes in our model, we include this effect through the IL-6 pathway. In other words, we assume hypoxia promotes IL-6 production by macrophages, which has a strong pro-inflammatory effect.

In this paper, we assume blood vessel formation is proportional to the number of endothelial cells (ECs), facilitating oxygen transportation. Thus, oxygen serves as a source dependent on ECs. Regions with oxygen levels below a critical threshold are considered hypoxic. We specify two hypoxic states: mild hypoxia initiating the HIF pathways (between 0.1% and 1% oxygen) and severe hypoxia causing cell cycle arrest (below 0.1%). Cells can die through mechanisms in the original model (via inhibitors or normoxic death) or undergo hypoxic death. To capture the promotion or inhibition effects of hypoxia, we define the following function:


(2.1) $$f_{i}(x)=\frac{(Ox_{crit}^{\left(i\right)}{)}^{m}}{(Ox_{crit}^{\left(i\right)}{)}^{m}+x^{m}},\;i=1,2$$

where $Ox_{crit}^{\left(i\right)}$ are the critical oxygen values and m is the Hill-like coefficient, which governs the decrease rate from the maximum to the minimum function value. Notice that two functions are defined in (2.1), corresponding to two critical oxygen values. The first critical value corresponds to the transitioning threshold from normoxia to mild hypoxia, and the second one marks the transition from mild to severe hypoxia.

#### 2.1.2. T-cells

We classify T-cells into naive ($T_{N}$), helper ($T_{h}$), cytotoxic ($T_{C}$) and regulatory ($T_{r}$) types. Activated subtypes differentiate from the naive subtype, introducing negative feedback terms proportional to their level and corresponding activator levels. These terms serve as positive feedback for the activated subtypes, indicating regulation by the available number of naive subtypes. We employ a similar method for naive and activated dendritic cells and macrophages.

We consider a constant intrinsic proliferation rate for naive T-cells. Naive T-cells diminish due to differentiation or normoxic and hypoxic cell death.

Helper T-cells’ differentiation from naive T-cells is promoted by HMGB1 [53,54], dendritic cells [55,56] and IL-12 [57,58]. They are inhibited by regulatory T-cells [59,60] and IL-10 [61,62]. We also consider normoxic and hypoxic cell death for helper T-cells.

Similarly, cytotoxic T-cells are promoted by dendritic cells [63,64] and IL-12 [58,65]. They are inhibited by regulatory T-cells [59,66] and IL-10 [65,67]. We consider normoxic and hypoxic cell death for these T-cells, too.

Like the other types, regulatory T-cell differentiation is promoted by dendritic cells [68,69]. Also, as mentioned in §2.1.1, mild hypoxia can increase regulatory T-cell differentiation. Similarly to the case of the other subtypes, we close the ODE by the normoxic and hypoxic cell death.

Note that the hypoxic cell death is modelled via $f_{2}$, but the hypoxic promotion of $T_{r}$ is modelled via $f_{1}$, corresponding to severe and mild hypoxia, respectively. The corresponding ODEs in this case read:


(2.2) $$\begin{array}{ll} \frac{d[\bar{T_{N}}]}{dt}\;\;\;\;\; & ={\bar{A}}_{T_{N}}\\ & -\left({\bar{\lambda }}_{T_{h}H}[\bar{H}]+{\bar{\lambda }}_{T_{h}D}[\bar{D}]+{\bar{\lambda }}_{T_{h}IL_{12}}[\bar{IL_{12}}]\right)[\bar{T_{N}}]\\ & -\left({\bar{\lambda }}_{T_{c}D}[\bar{D}]+{\bar{\lambda }}_{T_{c}IL_{12}}[\bar{IL_{12}}]\right)[\bar{T_{N}}]\\ & -\left({\bar{\lambda }}_{T_{r}D}[\bar{D}]+{\bar{\lambda }}_{T_{r}Ox}f_{1}([\bar{Ox}])\right)[\bar{T_{N}}]\\ & -\left(\delta_{T_{N}}+{\bar{\delta }}_{T_{N}Ox}f_{2}([\bar{Ox}])\right)[\bar{T_{N}}], \end{array}$$

(2.3) $$\begin{array}{ll} \;\;\frac{d[\bar{T_{h}}]}{dt}\;\;\;\;\; & =X\left({\bar{\lambda }}_{T_{h}H}[\bar{H}]+{\bar{\lambda }}_{T_{h}D}[\bar{D}]+{\bar{\lambda }}_{T_{h}IL_{12}}[\bar{IL_{12}}]\right)[\bar{T_{N}}]\\ & -\left({\bar{\delta }}_{T_{h}T_{r}}[\bar{T_{r}}]+{\bar{\delta }}_{T_{h}IL_{10}}[\bar{IL_{10}}]\right)[\bar{T_{h}}]\\ & -\left(\delta_{T_{h}}+{\bar{\delta }}_{T_{h}O_{x}}f_{2}([\bar{O_{x}}])\right)[\bar{T_{h}}], \end{array}$$

(2.4) $$\begin{array}{ll} \frac{d[\bar{T_{c}}]}{dt}\;\;\;\;\; & =\left({\bar{\lambda }}_{T_{c}D}[\bar{D}]+{\bar{\lambda }}_{T_{c}IL_{12}}[\bar{IL_{12}}]\right)[\bar{T_{N}}]\\ & -\left({\bar{\delta }}_{T_{c}T_{r}}[\bar{T_{r}}]+{\bar{\delta }}_{T_{c}IL_{10}}[\bar{IL_{10}}]\right)[\bar{T_{c}}]\\ & -\left(\delta_{T_{c}}+{\bar{\delta }}_{T_{c}O_{x}}f_{2}([\bar{O_{x}}])\right)[\bar{T_{c}}], \end{array}\;$$

(2.5) $$\begin{array}{ll} \frac{d[\bar{T_{r}}]}{dt}\;\;\;\;\; & =\left({\bar{\lambda }}_{T_{r}D}[\bar{D}]+{\bar{\lambda }}_{T_{r}O_{x}}\;f_{1}([\bar{O_{x}}])\right)\;[\bar{T_{N}}]\\ & -\left(\delta_{T_{r}}+{\bar{\delta }}_{T_{r}O_{x}}f_{2}([\bar{O_{x}}])\right)[\bar{T_{r}}]. \end{array}\;$$

#### 2.1.3. Dendritic cells

We model two dendritic cell types: naive ($D_{N}$) and activated (D) dendritic cells. Like T-cells, the activated dendritic cells differentiate from the naive ones. We also include normoxic and hypoxic cell death for both subtypes. Like naive T-cells, we consider a constant intrinsic growth rate for the naive dendritic cells. Their number decreases as they get activated or die. The tumour burden produces danger signals that promote dendritic cell maturation [70]. HMGB1 is another factor contributing to dendritic activation [71,72] and so is hypoxia, as explained in §2.1.1. Despite increasing the dendritic cell maturation, cancer cells can release factors inhibiting dendritic cell maturation and function [69].


(2.6) $$\begin{array}{ll} \frac{d[\bar{D_{N}}]}{dt}\;\;\;\;\; & ={\bar{A}}_{D_{N}}\\ & -\left({\bar{\lambda }}_{DC}[\bar{C}]+{\bar{\lambda }}_{DH}[\bar{H}]+{\bar{\lambda }}_{DO_{x}}f_{1}([\bar{O_{x}}])\right)[\bar{D_{N}}]\\ & -\left(\delta_{D_{N}}+{\bar{\delta }}_{D_{N}O_{x}}f_{2}([\bar{O_{x}}])\right)[\bar{D_{N}}], \end{array}$$

(2.7) $$\begin{array}{ll} \frac{d[\bar{D}]}{dt}\;\;\;\;\; & =\left({\bar{\lambda }}_{DC}[\bar{C}]+{\bar{\lambda }}_{DH}[\bar{H}]+{\bar{\lambda }}_{DO_{x}}f_{1}([\bar{O_{x}}])\right)[\bar{D_{N}}]\\ & -\left({\bar{\delta }}_{DC}[\bar{C}]+\delta_{D}+{\bar{\delta }}_{D_{N}O_{x}}f_{2}([\bar{O_{x}}])\right)[\bar{D}]. \end{array}$$

#### 2.1.4. Macrophages

We consider naive ($M_{N}$) and activated macrophages (M) in the model. For simplicity, the different activated macrophages, such as M1 and M2 types, are consolidated as M. Differentiation of these types along the lines (e.g. see [73]) would be an interesting topic for further study. Furthermore, we do not include hypoxic cell death for macrophages. Since these cell types tend to accumulate in hypoxic regions, they quickly adapt to severe hypoxia [74]. Like the other naive cell types, we model naive macrophages to proliferate at an innate constant rate. Their activation is augmented by IL-10 [75,76], IL-12 [65,77] and helper T-cells [78]. Both macrophage subtypes die via normoxic cell death. Activated macrophage polarity is defined through its interaction with cancer cells, which is why they are often referred to as anti (M1) and pro (M2) tumour macrophages [79]. However, as mentioned above, we do not explicitly include these subtypes in our model, and their different interaction with tumour cells is modelled through their cytokine pathways.


(2.8) $$\begin{array}{ll} \frac{d[\bar{M_{N}}]}{dt}\;\;\;\;\; & ={\bar{A}}_{M_{N}}-({\bar{\lambda }}_{MIL_{10}}[\bar{IL_{10}}]+{\bar{\lambda }}_{MIL_{12}}[\bar{IL_{12}}]\\ & +{\bar{\lambda }}_{MT_{h}}[\bar{T_{h}}]+\delta_{M_{N}})[\bar{M_{N}}], \end{array}$$

(2.9) $$\begin{array}{ll} \frac{d[\bar{M}]}{dt}\;\;\;\;\; & =({\bar{\lambda }}_{MIL_{10}}[\bar{IL_{10}}]+{\bar{\lambda }}_{MIL_{12}}[\bar{IL_{12}}]\\ & +{\bar{\lambda }}_{MT_{h}}[\bar{T_{h}}])[\bar{M_{N}}]-\delta_{M}[\bar{M}]. \end{array}\;$$

#### 2.1.5. Cancer and necrotic cells

Cancer cells (C) grow according to a logistic model with carrying capacity $C_{0}$. Many factors can promote cancer proliferation, among which we have explicitly included the IL-6 [80,81] and adipocyte effects [82,83]. The other factors are considered via a constant rate $\lambda_{C}$. Cancer cells can be cleared via cytotoxic cells [84,85]. Also, as mentioned, T-cell cytotoxicity increases through HIF pathways, which is modelled by the term including f1([Ox]). Finally, we include hypoxic and normoxic cell death mechanisms. Even though cancer cells proliferate much faster than normal cells, factors such as physical space, fitness landscapes and nutrition impose a carrying capacity on their growth [86,87]. We apply the same rationale to the other two cell types (described in the following sections), namely adipocytes and ECs.

A portion of cancer cell death turns into necrotic cells [88]. That portion is modelled via the constant αNC. We only consider regular cell clearance for necrotic cells since they are dead cells and do not go through hypoxia.


(2.10) $$\begin{array}{ll} \frac{d[\bar{C}]}{dt}\;\;\;\;\; & =\left(\lambda_{C}+{\bar{\lambda }}_{CIL_{6}}[\bar{IL_{6}}]+{\bar{\lambda }}_{CA}[\bar{A}]\right)\left(1-\frac{\left[\bar{C}\right]}{\bar{C_{0}}}\right)[\bar{C}]\\ & -\left({\bar{\delta }}_{CT_{c}}[\bar{T_{c}}]+{\bar{\delta }}_{CT_{c}O_{x}}f_{1}([\bar{O_{x}}])[\bar{T_{c}}]\right)[\bar{C}]\\ & -\left({\bar{\delta }}_{CO_{x}}f_{2}([\bar{O_{x}}])+\delta_{C}\right)[\bar{C}], \end{array}$$

(2.11) $$\begin{array}{ll} \frac{d[\bar{N}]}{dt}\;\;\;\;\; & ={\bar{\alpha }}_{NC}({\bar{\delta }}_{CT_{c}}[\bar{T_{c}}]+{\bar{\delta }}_{CT_{c}O_{x}}f_{1}([\bar{O_{x}}])[\bar{T_{c}}]\\ & +{\bar{\delta }}_{CO_{x}}f_{2}([\bar{O_{x}}])+\delta_{C})[\bar{C}]-\delta_{N}[\bar{N}]. \end{array}\;$$

#### 2.1.6. Adipocytes

Adipocytes contribute to cancer progression through intricate mechanisms. Having that contribution included in (2.10) as λCA[A], for simplicity, we model the adipocytes dynamics through a simple logistic model.


(2.12) $$\begin{array}{ll} \frac{d[\bar{A}]}{dt}\;\;\;\;\; & =\lambda_{A}\left(1-\frac{\left[\bar{A}\right]}{\bar{A_{0}}}\right)[\bar{A}]-\delta_{A}[\bar{A}]. \end{array}$$

#### 2.1.7. Cytokines

HMGB1 is produced by dendritic cells, necrotic cells, macrophages, cytotoxic T-cells and cancer cells [72,89]. IL-12 is secreted by dendritic cells and macrophages [90]. IL-10 is a product of dendritic cells, macrophages, cancer cells and cytotoxic, helper and regulatory T-cells [90]. Finally, adipocytes, dendritic cells and macrophages are the main IL-6 producers [91,92]. Note that we have a hypoxic promotion of IL-6 production by macrophages. As mentioned in §2.1.1, this is to model the increase in pro-tumour macrophage differentiation via mild hypoxia. Additionally, IL-10 and IL-6 increase through HIF pathways, as explained in §2.1.1. We include a natural decay term for cytokine clearance.


(2.13) $$\;\begin{array}{ll} \frac{d[\bar{H}]}{dt}\;\;\;\;\; & ={\bar{\lambda }}_{HD}[\bar{D}]+{\bar{\lambda }}_{HN}[\bar{N}]+{\bar{\lambda }}_{HM}[\bar{M}]+{\bar{\lambda }}_{HT_{c}}[\bar{T_{c}}]\\ & +{\bar{\lambda }}_{HC}[\bar{C}]-\delta_{H}[\bar{H}], \end{array}$$

(2.14) $$\begin{array}{ll} \frac{d[\bar{IL_{12}}]}{dt}\;\;\;\;\; & ={\bar{\lambda }}_{IL_{12}M}[\bar{M}]+{\bar{\lambda }}_{IL_{12}D}[\bar{D}]-\delta_{IL_{12}}[\bar{IL_{12}}], \end{array}$$

(2.15) $$\begin{array}{ll} \frac{d[\bar{IL_{10}}]}{dt}\;\;\;\;\; & ={\bar{\lambda }}_{IL_{10}M}[\bar{M}]+{\bar{\lambda }}_{IL_{10}D}[\bar{D}]+{\bar{\lambda }}_{IL_{10}T_{r}}[\bar{T_{r}}]\\ & +{\bar{\lambda }}_{IL_{10}T_{h}}[\bar{T_{h}}]+{\bar{\lambda }}_{IL_{10}T_{c}}[\bar{T_{c}}]+{\bar{\lambda }}_{IL_{10}C}[\bar{C}]\\ & +{\bar{\lambda }}_{IL_{10}O_{x}}f_{1}([\bar{O_{x}}])-\delta_{IL_{10}}[\bar{IL_{10}}], \end{array}$$

(2.16) $$\;\begin{array}{ll} \frac{d[\bar{IL_{6}}]}{dt}\;\;\;\;\; & ={\bar{\lambda }}_{IL_{6}A}[\bar{A}]+{\bar{\lambda }}_{IL_{6}M}[\bar{M}]+{\bar{\lambda }}_{IL_{6}MO_{x}}[\bar{M}]f_{1}([\bar{O_{x}}])\\ & +{\bar{\lambda }}_{IL_{6}D}[\bar{D}]+{\bar{\lambda }}_{IL_{6}O_{x}}f_{1}([\bar{O_{x}}])-\delta_{IL_{6}}[\bar{IL_{6}}]. \end{array}$$

#### 2.1.8. Newly added compartments: ECs, VEGF and oxygen

ECs proliferate on their own at a very slow rate [93]. However, with VEGF present, this growth rate increases significantly [94,95]; so, we utilize a VEGF-catalyzed logistic growth for their population. By contrast, endogenous angiogenesis inhibitors, such as IL-12, hinder EC’s proliferation [96]. There are other TAFs and endogenous angiogenesis inhibitors, but we only include VEGF and IL-12 here for simplicity. We consider normoxic and hypoxic cell death for ECs. Macrophages, adipocytes and hypoxic cancer cells secrete VEGF [95,97,98]. Finally, the ECs, which form vessels, will be the oxygen source, and all the cells consume oxygen. The consumption rates are grouped by cell type to avoid complexity.


(2.17) $$\begin{array}{ll} \frac{d[\bar{E_{N}}]}{dt}\;\;\;\;\; & ={\bar{\lambda }}_{VE_{N}}[\bar{V}](1-\frac{\left[\bar{E_{N}}\right]}{{\bar{E_{N}}}_{0}})[\bar{E_{N}}]-{\bar{\delta }}_{E_{N}IL_{12}}[\bar{IL_{12}}][\bar{E_{N}}]\\ & -(\delta_{E_{N}}+{\bar{\delta }}_{E_{N}O_{x}}f_{2}([\bar{O_{x}}]))[\bar{E_{N}}], \end{array}$$

(2.18) $$\begin{array}{ll} \frac{d[\bar{V}]}{dt}\;\;\;\;\; & ={\bar{\lambda }}_{VA}[\bar{A}]+{\bar{\lambda }}_{VM}[\bar{M}]+{\bar{\lambda }}_{VCO_{x}}[\bar{C}]f_{1}(Ox)\\ & +{\bar{\lambda }}_{VO_{x}}f_{1}([\bar{O_{x}}])-\delta_{V}[\bar{V}], \end{array}\;$$

(2.19) $$\;\begin{array}{ll} \frac{d[\bar{Ox}]}{d\tau }\;\;\;\;\; & ={\bar{\lambda }}_{O_{x}E_{N}}([\bar{E_{N}}])-{\bar{\delta }}_{1}\left(\left[\bar{T_{N}}]+[\bar{T_{h}}]+[\bar{T_{c}}]+[\bar{T_{r}}\right]\right)[\bar{O_{x}}]\\ & -{\bar{\delta }}_{2}\left(\left[\bar{D_{N}}]+[\bar{D}\right]\right)[\bar{O_{x}}]-{\bar{\delta }}_{3}\left(\left[\bar{M_{N}}]+[\bar{M}\right]\right)[\bar{O_{x}}]\\ & -{\bar{\delta }}_{4}[\bar{C}][\bar{O_{x}}]-{\bar{\delta }}_{5}[\bar{A}][\bar{O_{x}}]-{\bar{\delta }}_{6}[\bar{E_{N}}][\bar{O_{x}}]-\delta_{O_{x}}[\bar{O_{x}}]. \end{array}$$

### 2.2. Data preparation

We utilized RNA-sequencing data for three mice, accessible through the Gene Expression Omnibus (GEO) database under the accession code GSE76772 [36], encompassing four distinct tumour progression stages: hyperplasia at week 6, adenoma/MIN at week 8, early carcinoma at week 10 and late carcinoma at week 12. This data was primarily used to compare gene expression patterns across various cancer stages. Raw gene expression data was obtained through directional RNA-sequencing, and statistical techniques were employed to eliminate genes with low transcriptional activity. Then the refined dataset was normalized through differential expression analysis for sequence count data (DESeq) [99].

We use a digital cytometry method of CIBERSORTx B-mode [37] to infer the different cell fractions from the RNA-sequencing data. For this task, we need a signature matrix containing information about cell-specific gene expression signatures [100,101]. CIBERSORTx has an in-built signature matrix for Homo sapiens, but we need one for mice. So, we use the ImmGen signature matrix from the Immunological Genome Project [38]. It contains gene expression information for 207 immune cell types, most are certain immune cell subtypes consolidated as one variable in our model. For non-immune cells, a good resource is the Tabula Muris database [39]. They have a single-cell RNA-sequence (scRNA-seq) atlas for all important mouse organs and tissues. This data consists of two sets: data acquired from fluorescence-activated cell sorting (FACS) and data acquired from droplets. Based on the above paper, the former shows a better variety and is compatible with Immgen and CIBERSORTx. However, unlike the ImmGen data, this data cannot be readily used as a signature matrix. First, we must cross-reference the scRNA-seq count data with the FACS annotations to convert the obscure cell barcodes to meaningful cell names. Then, we upload our polished scRNA-seq data to CIBERSORTx to create a signature matrix. This will create a signature matrix to infer the non-immune cell fractions from the gene expression data.

We need a framework to estimate the number of cells from the calculated cell fractions. Sun *et al*. share their procedure of isolating macrophages and cancer cells for mouse breast cancer in a detailed protocol [102]. Their samples are derived from the same mouse model used in the study from which we obtained our data. According to their protocol, they can extract $4.5\pm 0.9\times 10^{7}$ cancer cells from 1 g of tumour with more than 90% precision. This number is obtained from two 0.5 g tumours, each almost 10 mm in diameter. They mention that they avoid collecting necrotic cells. They also collected $2.2\pm 0.6\times 10^{6}$ tumour-associated macrophages (TAMs) from 1 g of the same tumour with the same precision. In a study serving as the basis for this protocol, Sun *et al*. also provide information on tumour volumes at various stages [103]. According to their paper, the tumour volume measures approximately 200 $mm^{3}$ during hyperplasia, 400 $mm^{3}$ during adenoma, 700 $mm^{3}$ during early carcinoma and 1400 $mm^{3}$ during late carcinoma. These measurements are calculated in caliper format $L\times W^{2}/2$, where *L* is the long diameter and *W* is the short diameter. Assuming the tumour sections are close to spheres, we can take *L* to be approximately equal to *W*. Hence, during hyperplasia, the tumour is 7.36 mm in diameter, 9.28 mm during adenoma, 11.18 mm during early carcinoma and 14.09 mm during late carcinoma.

So if every 1 g sample requires two tumours of almost 10 mm diameter to get $4.5\pm 0.9\times 10^{7}$ cancer cells and $2.2\pm 0.6\times 10^{6}$ TAMs, we will have the following estimates for our data:

— $1.65\pm 0.33\times 10^{7}$ cancer cells and $8.09\pm 0.22\times 10^{5}$ TAMs during hyperplasia.
— $2.08\pm 0.41\times 10^{7}$ cancer cells and $1.02\pm 0.27\times 10^{6}$ TAMs during adenoma/MIN.
— $2.51\pm 0.50\times 10^{7}$ cancer cells and $1.22\pm 0.33\times 10^{6}$ TAMs during early carcinoma.
— $3.17\pm 0.63\times 10^{7}$ cancer cells and $1.54\pm 0.42\times 10^{6}$ TAMs during late carcinoma.

In this study, the number of TAMs adds up to $M_{N}+M$ from (2.8) and (2.9). With the cell fraction values available (figure 2) and knowing the total number of macrophages, we can promptly calculate the other immune cell quantities.

As for the non-immune cells, we use:


(2.20) $$\sum_{i\in I}N_{i}V_{i}+\sum_{j\in J}M_{j}V_{j}+N_{C}V_{C}=\text{Total volume}.$$

Where $N_{i}$ and $M_{j}$ are the numbers of i-th immune cell and j-th non-immune cell, respectively. The sets I and J contain indices for immune and non-immune cells (adipocytes and ECs). The values $V_{i}$ and $V_{j}$ are the single cell volume of the corresponding immune and non-immune cells. The values $N_{C}$ and $V_{C}$ are the number and volume of cancer cells. Recall that the reported tumour volumes are devoid of necrotic cells. Using table 2 with the tumour volumes mentioned, we can get the number of non-immune cells using formula (2.20).

**Table 2.** Size of different cell types in mice. We use the midpoint in our calculations for the lengths with a given range.

cell type	length^a^ (μm)	reference
dendritic cells	10–15	Tasnim *et al*. [104]
macrophages	21	Chitu *et al*. [105]
granulocytes and monocytes	13–15	Kornmann *et al*. [106]
lymphocytes (i.e. T-cells, B-cells and NKs)	10–15	O’Connell *et al*. [107]
stem cells	7–17	Pillarisetti *et al*. [108]
ECs	20	Jiang *et al*. [109]
luminal epithelial cells of mammary gland	10–50	Chen *et al*. [110]
cancer cells	12	Laget *et al*. [111]
adipose-tissue derived stem cells	28	Hoogduijn *et al*. [112]
fibroblasts^b^	10–20	Turgay *et al*. [113]
adipocytes	20	Hagberg *et al*. [114]

^a^ We assume the major (*L*) and minor (*W*) diameter of the cells are roughly equal and use the caliper format $L\times W^{2}/2(=L^{3}/2)$ to get the volume.

^b^ We have not imputed cell fractions for fibroblasts. However, since most mammary stromal cells are adipocytes and fibroblasts, we use this fact to take the intersection of their approximate size (20 μm) as the size of stromal cells.

Tables 3 and 4 show the calculated number of cells along with the value of cytokines inferred directly from the gene expression data. For the oxygen levels, Ebbesen *et al*. report the following [44]:

— 10 000–50 000 ppm is normoxic (equivalent to 1–5% *$O_{2}$* per kPa).
— 1000–10 000 ppm is mild hypoxia when HIF pathways become significant (equivalent to 0.1–1% $O_{2}$ per kPa).
— 10–1000 ppm is severe hypoxia when cell death or cell cycle arrest is triggered (equivalent to 0.001–0.1% $O_{2}$ per kPa).

**Table 3.** Number of cells.The numbers in each cell are integer cutoffs of the calculated values.

sample: stage	$T_{N}$	$T_{h}$	$T_{C}$	$T_{r}$	$D_{N}$	D	$M_{N}$	M	C	N ^a^	A	$E_{N}$
M1: W6	13 82 527	6 63 086	2 28 615	92 289	12 27 231	8 86 534	3 89 295	3 97 821	1 32 00 000	66 00 000	4 16 120	9 15 268
M1: W8	16 15 524	6 41 814	2 65 967	1 76 412	15 74 935	10 57 404	5 77 007	7 10 944	1 67 00 000	83 50 000	15 82 762	9 16 916
M1: W10	25 69 211	6 89 479	3 10 204	2 92 578	16 36 578	13 28 095	6 12 205	9 42 441	2 01 00 000	1 00 50 000	27 63 480	7 69 340
M1: W12	21 99 247	9 37 204	2 22 401	1 47 089	23 24 486	16 86 078	7 87 834	11 71 025	2 54 00 000	1 27 00 000	52 22 452	30 28 739
M2: W6	14 41 964	5 29 513	1 28 158	79 395	11 15 894	9 34 727	4 50 621	3 58 170	1 65 00 000	82 50 000	4 61 103	4 93 492
M2: W8	15 45 885	5 83 058	5 89 238	4 07 590	11 52 659	14 11 717	2 92 503	4 55 659	2 08 00 000	1 04 00 000	10 80 624	1 85 467
M2: W10	13 11 444	4 78 978	1 83 104	69 789	12 43 110	8 59 137	4 30 970	4 58 725	3 01 00 000	1 50 50 000	24 64 786	9 31 836
M2: W12	22 97 591	4 26 244	1 27 776	1 81 325	13 96 820	10 57 303	5 59 029	5 62 339	3 17 00 000	1 58 50 000	55 78 533	14 69 427
M3: W6	13 99 198	4 30 269	4 05 214	79 316	10 21 227	9 24 575	3 95 039	4 35 495	1 98 00 000	99 00 000	3 33 365	3 66 687
M3: W8	14 67 427	5 07 931	3 95 476	81 323	12 68 459	8 36 283	4 39 574	5 75 209	2 49 00 000	1 24 50 000	15 14 664	7 85 294
M3: W10	21 61 705	3 40 691	2 72 777	1 88 039	12 32 740	13 46 494	5 54 893	6 64 238	2 51 00 000	1 25 50 000	28 85 384	11 96 948
M3: W12	31 71 264	9 63 737	3 81 975	1 62 666	19 09 543	11 77 989	6 58 718	8 84 036	3 80 00 000	1 90 00 000	54 37 350	29 37 968

^a^ We assume the number of necrotic cells is half of cancer cells. This is a crude approximation based on observations from the literature about the percentage of viable space in the TME occupied by necrotic cells [115].

**Table 4.** Cytokine levels. All the levels are, in fact, gene expression levels except for oxygen. The unit for oxygen concentration is mol/l.

sample: stage	H	$I\,L_{12}$	$I\,L_{10}$	$I\,L_{6}$	V	O⁢x
M1: W6	1000	63	417	384	776	3.125
M1: W8	940	41	351	197	536	0.625
M1: W10	1103	3	404	184	494	0.0625
M1: W12	1050	2	455	193	798	0.000625
M2: W6	1182	0	450	196	775	3.125
M2: W8	932	16	723	35	721	0.625
M2: W10	945	0	429	162	313	0.0625
M2: W12	807	4	319	127	384	0.000625
M3: W6	1521	27	511	336	1081	3.125
M3: W8	1549	22	566	312	1054	0.625
M3: W10	957	3	349	229	565	0.0625
M3: W12	779	10	278	163	909	0.000625

Noting the oxygen molar mass is 16 g/mol, we can turn the above values into mol/l values. Cai *et al*., whose data we use, speculate that the decrease in the expression of tricarboxylic acid cycle (TCA cycle) genes in later cancer stages is due to hypoxia. Therefore, the levels given for oxygen in table 4 are picked such that the later stages go through hypoxic conditions.

All the data is non-dimensionalized before associating them with our model. The non-dimensionalization is done by dividing each cell or cytokine value in tables 3 and 4 by the largest value of that cell or cytokine across all samples and all stages. Also, in simulations, we assume our week 6 is the system’s initial state, corresponding to t=0.

### 2.3. Parameter estimation

We use an HGA scheme based on natural evolution for parameter estimation [116]. We utilize MATLAB’s HGA, which is included in the Global Optimization Toolbox. First, we initialize a loss function that calculates the distance between the predicted values and the measured data.

We then start with a randomly produced set of parameters called the initial population. This can be constrained, biased or a free random pick. For our problem, we do not accept negative parameter values. However, after several tests, we saw that the algorithm does not produce parameter values bigger than 2 for large upper bounds. So, we take the upper bound to be 2.

In each iteration, the algorithm leverages the individuals in the present generation to construct the succeeding population. The creation of this new population involves the following steps:

— Assessing each member’s fitness in the current population yields the raw fitness scores. This is done by utilizing the loss function.
— Scaling these raw fitness scores into a more practical range of values, referred to as expectation values.
— Selecting parents and individuals chosen based on their expectation values. MATLAB’s default selection method constructs a line where each parent is associated with a line segment. The scaled value of the respective parent determines the length of each segment. The algorithm advances along the line in uniform steps, and at each step, it assigns a parent from the segment it arrives at. This way, we can pick out the elite children.
— Identifying certain individuals in the current population with lower fitness as elite members to be carried over to the next population.
— Generating offspring from the selected parents through random alterations to a single parent (mutation) or combining the attributes of a pair of parents (crossover).
— Substituting the existing population with the newly created offspring to constitute the subsequent generation.
— The algorithm stops when one of the stopping criteria is met.
— A local search algorithm starts using the best solution found by the genetic algorithm as the initial guess. We use the widely known MATLAB function fmincon, a gradient-based nonlinear constrained local minimizer.

The last step is only adopted in HGA. The choice of the local minimizer depends on the problem. For the theory behind the genetic algorithm, we refer the readers to Holland’s book [117]. Also, for a review of the genetic algorithms and different hybrid versions, see the article by El-Mihoub *et al*. [116].

Table S1 in the electronic supplementary material shows the non-dimensional estimated values of the parameters for mice 1, 2 and 3. As mentioned, non-dimensionalization is done by scaling each cell and cytokine level by the highest value across all three mice and all time points. This will enable us to compare the three mice and make the parameter estimation more stable. Note that we do not non-dimensionalize the time scale.

To summarize, we have 18 state variables and equations and 86 parameters. Using the available RNA-sequencing data for three MMTV-PyMT mice, we prepare data for all 18 variables at four essential time points: week 6, week 8, week 10 and 12. By fitting the equations to the acquired data through an HGA algorithm, we estimate the value of the parameters.

### 2.4. A discussion about the model’s identifiability

We recognize that the assumptions and connections we made with the literature to obtain the data may be optimistic. For example, the cell sizes we used to infer the number of non-immune cells can add unforeseen uncertainties to our findings. However, our framework does result in plausible values of cells and cytokines, and if all of the above can be reported under the same study, then this can be a meaningful starting point for further studies and refinements.

Assuming all the model variables are practical outputs (i.e. are available for all times), our model and its parameters are Full Input-State-Parameter Observable (FISPO), a term coined by Villaverde *et al*. [118]. One can evaluate the global structural identifiability of nonlinear systems by employing concepts from differential geometry. This assessment primarily relies on computing the rank of a specialized matrix known as the observability–identifiability matrix, formed by utilizing Lie derivatives. If the parameters are included in this computation as zero-dynamic variables, then the observability analysis of the parameters will be equivalent to their identifiability. For more details of these methods, see [118–122].

We use the MATLAB package STRIKE-GOLDD to ascertain the global structural identifiability of our model’s parameters. As previously noted, STRIKE-GOLDD declares all the model parameters structurally identifiable, assuming all our variables can be regarded as practical outcomes (i.e. data is available for them at all times).

**Figure 3.**
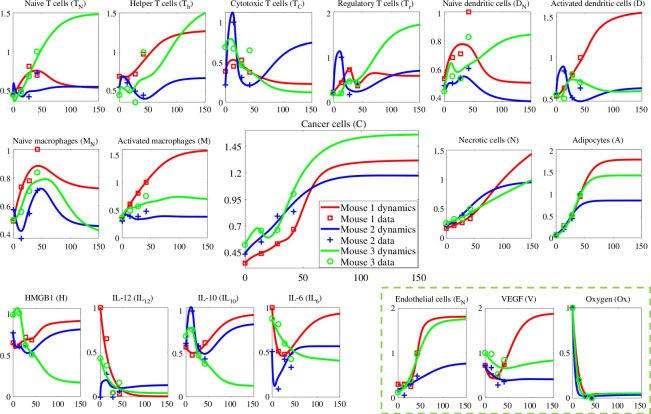
Cells and cytokine dynamics. The *x*-axes show time in days. The *y*-axes are the dimensionless levels. The figures in the box correspond to the model extension.

A parameter can be globally structurally identifiable but not practically identifiable [123]. A parameter that minimizes the loss function (or maximizes the likelihood function) but has an infinite confidence interval is considered a practically non-identifiable parameter [124]. To investigate these parameters within the estimated range, we create synthetic data by perturbing the solutions in figure 3 by 5%. Then we calculate the profile likelihood (see [124]) of each parameter, i.e. $PL(\theta_{i})$. This is done by keeping the corresponding $\theta_{i}$ fixed and minimizing the loss function with respect to the rest of the parameters ($\theta_{j}$ where j≠i). Next, we vary $\theta_{i}$ for each calculated profile likelihood and record the changes in the profile likelihood function. We then calculate a threshold for each set of estimated parameters by $L(𝜽)+\Delta_{\alpha }$, where L is the loss function used for parameter estimation and $\Delta_{\alpha }$ is the α-quantile of the $\chi^{2}$ distribution with df=1 for pointwise confidence intervals. Profile likelihoods not exceeding this threshold will be labelled as practically non-identifiable within the specified parameter range. Additionally, parameters with very flat profile likelihood functions may have local structural identifiability issues around the estimated value. Refer to figures S1–S3 in the electronic supplementary materials for results. Within the investigated parameter neighbourhoods ($\left[0,2*\theta_{i}\right]$), mouse 1 has 41 practically non-identifiable parameters, with 31 displaying visibly flat profiles, potentially indicating local structural non-identifiability. These numbers are lower for mice 2 and 3, which aligns with their Principle Component Analysis (PCA) count of sensitive variables. Mouse 2 and 3 have 35 parameters that are practically non-identifiable, with 12 from mouse 2 and 13 from mouse 3 having visibly flat profile likelihoods within the studied parameter range. This shows these parameters are likely to remain non-identifiable even with increased data points or measurement quality. Notably, none of these parameters with flat likelihood profiles are identified as sensitive parameters for each mouse, suggesting their values will not significantly impact system robustness. However, we acknowledge limitations in this approach, as it relies on synthetic data and assumes all variables are always observable. However, the method can be adapted to accommodate feasible experimental situations.

### 2.5. Sensitivity analysis

Our study employs a Principal Component Global Sensitivity Analysis (PCGSA) to scrutinize the parameter’s impact on our model. To accomplish this, we utilize the Matlab package PeTTSy. The methodology for calculating sensitivity measures is derived from the approach outlined by the PeTTSy developers in their seminal paper [125]. We provide a summary of their technique here. We strongly encourage readers to consult the original study for a more comprehensive understanding and in-depth insights, as our description serves only as an overview of their detailed method.

Suppose $X_{m}(t,𝐤)$ is a solution of an ODE system with t being the time variable and $𝐤=(k_{1},k_{2},\cdots \;,k_{N})$ being the parameters vector. So the *m*-th solution derivative with respect to the *j*-th parameter at a time t is given by $\partial X_{m}(t,k)/\partial k_{j}\;(t)$. A Singular Value Decomposition (SVD) analysis is carried out to calculate the global sensitivity. This is to investigate the linearization of the mapping from perturbed parameters $\delta 𝐤\in R^{N}$ to the changes they cause in the solution, namely δ𝐗. Notice the mapping is to an infinite dimensional space of smooth functions. PeTTSy approximates these functions by evaluating them on a fine time grid (with n time steps), which results in high-dimensional vectors. The final product will be a large matrix M=∂X/∂k with nK rows and N columns, where K is the number of our state variables. Like the state variables, we can include any other measurements, such as the sum of all the cells or the sum of all immune cells, as a single variable for the sensitivity analysis.

The issue is that this matrix is relatively large, and investigating the parameters effect on the time-variant of a large set of solutions can be tedious. This is where the SVD and PCA are helpful. They help us focus only on a handful of Principal Components (PCs) to understand the overall parameters effect on the whole model. We start with a singular value decomposition of M,


(2.21) $$M=U\Sigma V^{T},$$

where U is an nK×nK orthogonal matrix, Σ is an nK×N rectangular diagonal matrix with singular values $\sigma_{i}$ on its diagonal, and V is a matrix whose columns form an orthonormal basis for the parameter space. The principal global sensitivity values are $S_{ij}=\sigma_{i}V_{ji}$. They show


(2.22) $$\delta X=\sum_{i,j}S_{ij}\delta k_{j}\sigma_{i}U_{i}+O(||\delta k^{2}||),$$

where $\sigma_{1}\geq \sigma_{2}\geq \cdots \geq \sigma_{N}\geq 0$ are the singular values and $U_{i}$ (the columns of U) are PCs.

For the parameter sensitivity plots, we use the values $S_{ij}=\sigma_{i}V_{ji}$ for each PC $U_{i}$. To determine how many PCs are needed to get a good sense of the system’s overall sensitivity to parameters, we utilize a singular spectrum plot. We filter out the PCs corresponding to insignificant singular values.

We also investigate the sensitivity of cancer cells and the total number of immune cells to the model parameters. Given that we only get one significant singular value for these two cases, the PC sensitivity and the classical notion of sensitivity will be synonymous according to the relationship (2.22).

## 3. Results

### 3.1. Dynamics

We solve the non-dimensional ODEs (2.2)–(2.19) corresponding to figure 1 with parameters given in electronic supplementary materials (table S1). The results are shown in figure 3; the corresponding findings illustrate a good agreement with the data. The mouse data is cut off after 12 weeks due to the subject’s euthanizing, but we follow the dynamics until day 150. Moreover, the tumour samples are collected starting at week 6, which in the models marks t=0. Due to the system’s complexity, we comment primarily on the dynamics related to the model extension, namely the ones involving ECs, VEGF and oxygen. Further investigation will be carried out through sensitivity analysis.

Figure 3 shows a qualitative resemblance between the macrophage and VEGF dynamics. Mice 1 and 2 have the highest and lowest number of macrophages, respectively, consistent with adipocyte numbers, especially after t=40. Notice that this is true in numerical observations, even though not manifested in the experimental data for earlier times. The causal effect is expected as macrophages and adipocytes are primary VEGF producers in our model, besides hypoxic cancer cells and mild hypoxia-induced pathways. Initially, VEGF levels decrease due to higher oxygen levels delaying hypoxic cancer cells and other hypoxia mechanisms contributing to its production. However, despite this decrease, endothelial cells (ECs) increase in mice 2 and 3, attributed to lower initial IL-12 levels in these subjects compared with mouse 1. Consequently, even with a higher inhibition rate, ECs do not significantly decrease due to their low IL-12 levels.

Having the lowest endothelial cells (ECs), mouse 2 experiences the most rapid decline in oxygen levels, maintaining the lowest oxygen values throughout the simulation. However, during the time-period from 0 to 20, where oxygen decrease is significant, the blue curve representing $E_{N}$ for mouse 2 surpasses that of mouse 3. This phenomenon is attributed to IL-12 levels as discussed earlier. Despite starting with more oxygen producers ($E_{N}$), mouse 2 experiences a more pronounced oxygen decrease compared with mouse 3 due to a higher number of oxygen-consuming cell types during this short interval. In addition to ECs, mouse 2 has more T-cells, activated dendritic cells, naive macrophages and adipocytes during the initial time steps compared to mouse 3, see figure 3.

Mouse 3 has the most cancer cells, followed by mouse 1 for long enough times. This could be influenced by various molecular, cellular and hypoxic pathways, with cytotoxic T-cells enhanced by HIF pathways playing a significant role. Mouse 2, having the highest cytotoxic T-cells and the lowest adipocytes, shows the least cancer cell growth. The next section will explore more analysis of the key pathways through sensitivity analysis.

### 3.2. Sensitivity analysis results

**Figure 4.**
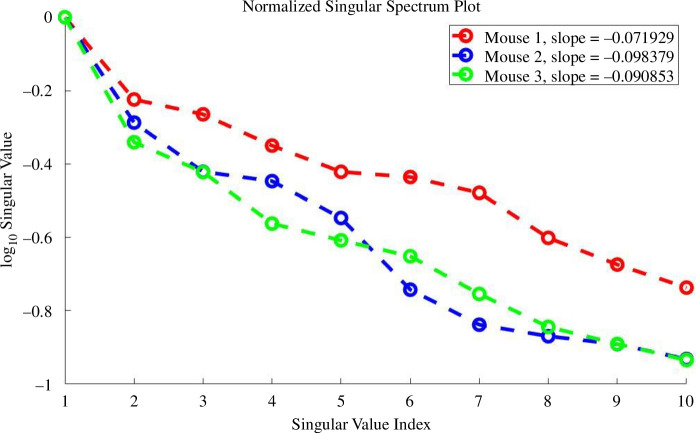
Singular spectrum. Top 10 largest singular values for mice 1, 2 and 3.

We analyse the sensitivity of the entire system, cancer cells and total immune cell count $\left(T_{N}+T_{h}+T_{C}+T_{r}+D_{N}+D+M_{N}+M\right)$ to model parameters. Due to numerous state variables, we conduct a PCGSA for the first analysis. Classical global sensitivity and first PC sensitivity are equivalent for the latter two. First, we conduct a comprehensive PCGSA of the entire system relative to its parameters. With 89 parameters and 18 variables, analysing all 18 PCs may be redundant. To determine the necessary PCs, we perform a singular spectrum analysis. Figure 4 displays each mouse’s top 10 normalized singular values. In mouse 1, the eighth singular value is approximately 20% of the first. For mice 2 and 3, this occurs around the fifth and sixth singular values, respectively. Therefore, we focus on sensitivity to variations in the top eight PCs for each mouse.

**Figure 5.**
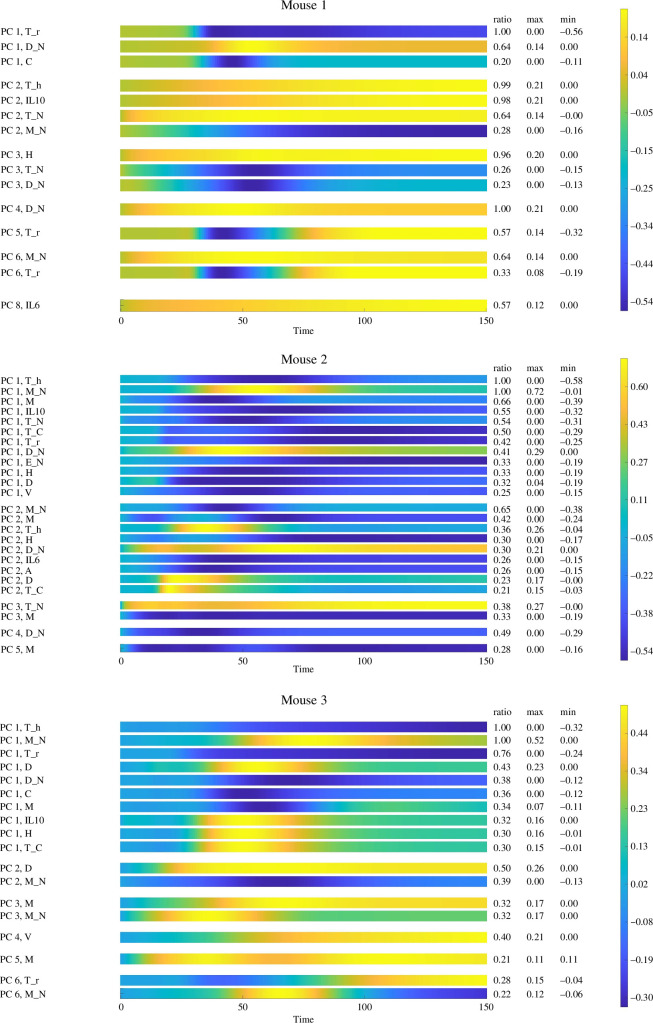
State variables sensitivity heat maps with respect to time. Only variables above 20% of the maximum global sensitivity are shown for each mouse. For each PC, the represented variables are sorted according to their scaled ratio of change.

Figure 5 shows state variables more sensitive to parameter variations when different PCs are considered, illustrating $\sigma_{i}U_{i}$ coefficients in (2.22). Only the variables whose sensitivity exceeds 20% of the global maximum sensitivity are included. There are sensitive state variables for all eight PCs considered in the analysis for mouse 1. However, mice 2 and 3 only have variables exceeding 20% of the global maximum up to the fifth and sixth PCs, respectively. This was expected based on the observations in figure 4. We notice the dominant presence of immune cells for each PC in all three subjects. Cells such as regulatory and helper T-cells, macrophages and dendritic cells are among the most repeated variables. These cells are all involved in producing IL-10, the most sensitive cytokine in the plot. This plot can also help in designing experiments for validation. The variables shown in this plot and, more specifically, the ones shared in all three subjects are appropriate choices for experimental observations.

**Figure 6.**
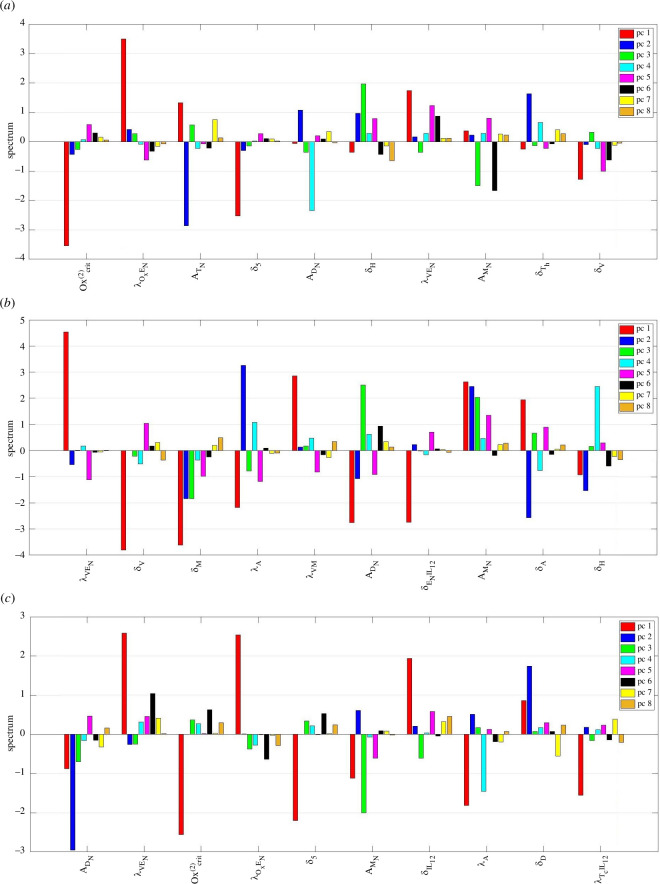
PCGSA of the top 10 most sensitive parameters for (*a*) mouse 1, (*b*) mouse 2 and (*c*) mouse 3.

Figure 6 shows the PCGSA result for mice 1, 2 and 3. This plot has to be accompanied by figure 5 for clearer biological inferences. Parameters present in all three plots are $\lambda_{VE_{N}}$, $A_{M_{N}}$ and $A_{D_{N}}$. Changing $\lambda_{VE_{N}}$ inflicts the highest variation in the first PC in all three mice. Figure 5 shows immune cells such as regulatory and helper T-cells, macrophages and dendritic cells are the most susceptible to this parameter variation. This parameter describes the promotion rate of EC proliferation by VEGF. One can connect this to the rate at which vessels are formed in the microenvironment. Also, $\lambda_{VE_{N}}$ involves two variables from the new model extension; see figure 1.

Parameters $A_{M_{N}}$ and $A_{D_{N}}$ are the innate naive macrophages and naive dendritic cell proliferation rates. For mouse 1, $A_{M_{N}}$ affects PC 6 and 3, with naive macrophages and HMGB1 receiving the highest impact. In mice 2 and 3, PCs 1, 2, 3 and 5 are affected the most, with naive and activated macrophages and helper and regulatory T-cells being the most sensitive variables to this parameter. Finally, besides the dendritic cell subtypes, helper and naive T-cells and activated and naive macrophages are included in the PCs, which are significantly influenced by $A_{D_{N}}$ variation. However, these effects are expected, as they control the production of two crucial naive immune cells and, consequently, their activated form. These cell types are involved in some of the most central interactions within the system.

Oxygen-related parameters $Ox_{crit}^{\left(2\right)}$, $\lambda_{OxE_{N}}$ and $\delta_{5}$ are among the top 10 most sensitive parameters in mice 1 and 3. All three show significant sensitivity for the first PC, greatly affecting helper and regulatory T-cells, macrophages and dendritic cells. The parameter $Ox_{crit}^{\left(2\right)}$ decides the transitioning threshold from mild to severe hypoxia, $\lambda_{OxE_{N}}$ describes how much oxygen is provided by ECs/vessels and $\delta_{5}$ is the oxygen uptake rate by adipocytes. This is interesting since in our other studies, parameters related to adipocytes had a considerable effect on the model [19,34]. Adipocytes are abundant in breast tissue, and adiposity is considered a well-known risk factor [126]. Besides affecting cancer directly, this suggests adipocytes also affect the microenvironment immune profile through oxygen-related pathways. Moreover, $\lambda_{A}$ and $\delta_{A}$, which are adipocyte production and death rates, are placed among sensitive parameters; see figure 6 and tables S2–S4 in the electronic supplementary material.

We should point out that IL-12 is affecting mouse 2’s first PC by inhibiting ECs ($\delta_{E_{N}IL_{12}}$). It has a similar effect on mouse 3 by promoting the cytotoxic cells ($\lambda_{T_{C}IL_{12}}$). So, despite not being among the sensitive state variables in figure 5, IL-12 bears a considerable impact on the immune profile through the PC1 sensitive cells such as $T_{h}$, $M_{N}$, M and $T_{r}$. Finally, in mouse 2, the VEGF production by macrophages ($\lambda_{VM}$) seems to have a similar effect through PC1 on the system.

**Figure 7.**
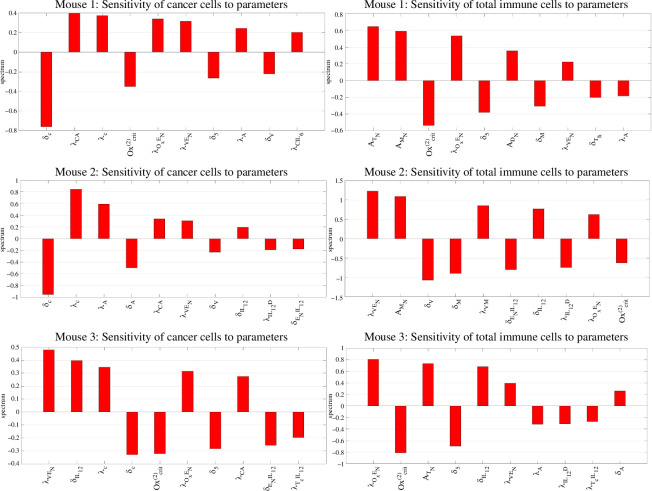
Sensitivity analysis with respect to cancer and total immune cells. Top 10 most sensitive parameters with respect to (left) cancer cells, (right) total immune cells.

Overall, the PCGSA shows the importance and intricacy of the immune system interactions in the TME. All parameter variations, especially parameters involving vasculature and oxygen delivery from our model extension, substantially influence crucial immune cells. We now examine whether the previously discussed parameters significantly influence diagnostic metrics, specifically the cancer and the total immune cell counts. Figure 7 illustrates the sensitivity analysis focusing on these crucial measures.

As expected, cancer levels are sensitive to parameters directly involved in the equation for their evolution (see 2.10) such as $\delta_{C}$, $\lambda_{C}$, $\lambda_{CA}$ and $\lambda_{CIL_{6}}$. This result agrees with the findings from the simpler model [34]. Also, when calculating the total immune cells’ sensitivity to parameters, immune cells’ innate production and death rates appear among the most sensitive parameters.

In figure 7, $\lambda_{VE_{N}}$, $Ox_{crit}^{\left(2\right)}$ and $\lambda_{OxE_{N}}$ are the most repeated, warranting particular attention in our study. They were present in the PCA and are, importantly, a part of the model extension; see figure 1. The results show that an increase in $\lambda_{VE_{N}}$ leads to an increase in all subjects’ immune cells and cancer cells. This might be due to less hypoxic death for cancer cells or more pro-tumour cytokine production by immune cells such as macrophages. Increasing $Ox_{crit}^{\left(2\right)}$ causes a quicker transition to severe hypoxia, decreasing the total number of immune and cancer cells. This effect can be due to several factors, such as an increase in cytotoxicity or a decrease in IL-6 production. Note that the result for cancer sensitivity to $Ox_{crit}^{\left(2\right)}$ is not included in figure 7 for mouse 2 since it is the 13th most sensitive parameter. Nevertheless, the value is negative like the others.

The oxygen delivery rate by the ECs/vessels ($\lambda_{OxE_{N}}$) is positively related to the total number of immune and cancer cells. Again, this is not included in the figure for mouse 2 since it is the 11th most sensitive parameter. The model extension effect on cancer cells through these parameters and the immune system showcases the importance of angiogenesis and oxygenation in the TME. This, in turn, introduces a limitation to our work regarding the cancer sensitivity to $Ox_{crit}^{\left(2\right)}$. Even though the hypoxic contribution to cancer proliferation through HIF pathways is included, this study considers purely oxidative phosphorylation for cells and not glycolysis as a metabolism approach. However, the metabolic switch to glycolysis under hypoxic stress is a common adaptive response from cancer cells and a challenge in cancer research [127]. Therefore, increasing $Ox_{crit}^{\left(2\right)}$ might not necessarily lead to fewer cancer cells if it induces a quicker metabolic change. These intricate mechanisms require deeper analysis and a more complicated model, which we defer to future research.

The relationship between adipocyte-related parameters and cancer and total immune cells can be explained by investigating the sensitivity value signs for $\lambda_{A}$, $\delta_{A}$, $\delta_{5}$ and $\lambda_{CA}$. In summary, if we increase the adipocyte number through increasing $\lambda_{A}$ or decreasing $\delta_{A}$, the total number of immune cells decreases, and of cancer cells increases. Increasing adipocytes greatly increases oxygen consumption, which is lethal for all cells affected by the hypoxic cell death, including immune cells. However, this effect is foiled for cancer cells by the direct adipocytes effect on cancer proliferation through $\lambda_{CA}$.

An increase in VEGF leads to more immune cells and more cancer cells. This can be done through decreasing $\delta_{V}$ (VEGF decay rate) in mice 1 and 2 and increasing $\lambda_{VM}$ (VEGF production by macrophages) in mouse 2. The same effects occur for mouse 3 but are not included among the top 10 in figure 7.

We also investigate the effect of IL-12-related parameters. Inhibiting the IL-12 production (increasing $\delta_{IL12}$) leads to more immune cells and cancer cells in mice 2 and 3. Less IL-12 means less cytotoxic T-cell activation, a major cancer cell inhibitor. Also, less IL-12 means more EC proliferation (see $\delta_{E_{N}IL_{12}}$), which results in more oxygen, promoting the proliferation of all immune and cancer cells. See the effect of its increased production by dendritic cells ($\lambda_{IL_{12}D}$) on cancer and immune cells in mice 2 and 3 in figure 7. Lastly, $\lambda_{T_{C}IL_{12}}$ is negatively associated with cancer cells and total immune cells in mouse 3. This is the cytotoxic T-cell activation rate by IL-12, one of the primary cancer cell inhibitors.

**Figure 8.**
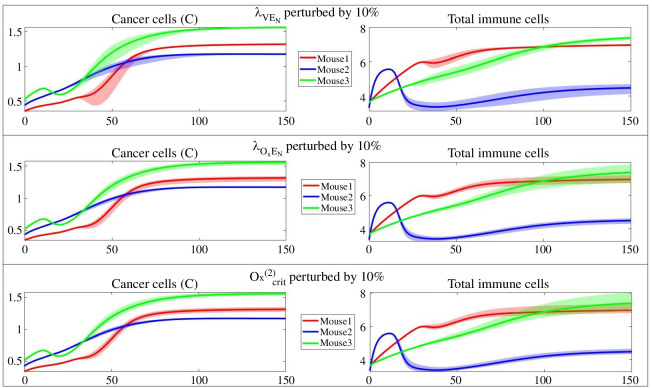
Effect of parameter perturbation on dynamics. The effect of perturbing $\lambda_{VE_{N}}$ (top), $\lambda_{OxE_{N}}$ (middle) and $Ox_{crit}^{\left(2\right)}$ (bottom) on dynamics of cancer cells and total immune cells.

We finally explore the effect of perturbing $\lambda_{VE_{N}}$, $Ox_{crit}^{\left(2\right)}$ and $\lambda_{OxE_{N}}$ on the cancer dynamics and total immune cells given their presence in all three sensitivity analyses for three mice. These parameters are associated with our model extension and include the effect of all three new compartments: ECs, VEGF and oxygen. Figure 8 shows the effect of the aforementioned parameters’ perturbation on cancer and total immune cell dynamics. For $\lambda_{VE_{N}}$, the largest impact is between t=30 and t=80, which wears off as cancer approaches a steady state. The impact becomes visible for the total immune cells between times 20 and 30. For mouse 1, it is a short-lived effect wearing off around time 70. For mouse 3, it takes longer but eventually tapers after time 100, and mouse 2 shows a consistent regime throughout the simulation time. This observation implies controlling cancer through controlling angiogenesis and VEGF inhibitors should happen in earlier stages to have a positive impact. Once cancer reaches its steady state, these variations do not leave a significant impact. Cancer steady-state translates into a state in which cancer cannot physically grow any further due to space or resources depletion. It has been discussed in the literature for humans and mice that VEGF therapy for late-stage breast cancer has not been correlated with better prognoses [128].

Perturbing $\lambda_{OxE_{N}}$ and $Ox_{crit}^{\left(2\right)}$ results in a sustainable but less significant effect. Especially in mice 1 and 3, the impact region for total immune cells increases in time. Even though controlling hypoxia or oxygen delivery is not medically feasible, numerous studies focus on targeting the HIF pathways for treatment [129–131].

## 4. Discussion

In this study, we extended a previously developed ODE model, as described in [34], to explore the mechanisms that drive or result from angiogenesis within the breast TME of PyMT-MMTV mice. We added three important elements: ECs, VEGF and oxygen. ECs, forming the blood vessels’ infrastructure, are crucial for transporting metabolites like oxygen. VEGF, a key cytokine, initiates and accelerates angiogenesis. It is secreted by various cells, including macrophages, adipocytes and hypoxic cancer cells. Additionally, ECs’ activity is modulated by IL-12. By incorporating these components, we effectively linked them with the previously developed model [34], thereby investigating the effect of angiogenic processes and hypoxia in breast TME.

Using PyMT-MMTV mice model data and HGA parameter estimation, we approximated the ODE model parameters and simulated results for 150 days. Significant differences emerged in dynamics: mouse 1 showed the highest cancer levels, while mouse 2 showed the lowest levels attributed to cytotoxicity and adiposity. Other notable interactions included high levels of helper T-cells and dendritic cells in mouse 1, linked to HMGB1, and elevated activated macrophages due to IL-10 and helper T-cells. Also, the number of ECs correlated with VEGF levels. However, some pathways could not be directly inferred from the interaction network, requiring global sensitivity analysis experiments for further investigation.

Through sensitivity analyses, we observed that similar to a previous study, adipocyte-related parameters (production and death rates) were crucial to the system [34]. Additionally, the oxygen consumption by adipocytes was found to be sensitive. Several studies have highlighted adipocytes’ effect on hypoxia in the TME. Nieman *et al*. argue that in individuals with excessive adipocytes, the adipose tissue is associated with chronic hypoxia, contributing to tumour progression through HIF pathways [132]. Trayhurn reasons the adipocytes’ sheer mass and abundance is a hypoxia-promoting factor [133].

Furthermore, a subset of parameters consistently emerged as influential factors affecting the whole system’s PCs and the dynamics of cancer cells and the immune system in all subjects. These parameters, which describe the ECs promotion by VEGF ($\lambda_{VE_{N}}$), oxygen delivery by the ECs/vessels ($\lambda_{OxE_{N}}$), and the threshold value for transitioning from mild to severe hypoxia ($Ox_{crit}^{\left(2\right)}$), are all related to angiogenesis and oxygenation. The first parameter introduces an intervention opportunity for controlling cancer progression and modulating the immune response. By contrast, $\lambda_{OxE_{N}}$ depends more on the cells’ fitness landscape and tissue content. In all three mice, increasing $\lambda_{VE_{N}}$ led to higher cancer cell counts and greater total immune cell numbers, suggesting angiogenesis plays a crucial role in the TME. Additionally, changing the threshold $Ox_{crit}^{\left(2\right)}$ influenced the immune cell dynamics and cancer progression. These findings underscore the importance of oxygen levels in the microenvironment and suggest targeting hypoxia-related pathways could be a promising avenue for therapy. Recall, however, that the cancer cell metabolic plasticity is a relevant factor [134] that merits further study. The oxygen delivery rate by ECs/vessels ($\lambda_{OxE_{N}}$) consistently impacted cancer and immune cells in all three mice. This shows the critical role of efficient oxygen transport in regulating the immune response within the TME. It also highlights the importance of angiogenesis in oxygen supply, which can affect both immune cell function and cancer cell proliferation.

We vary the three parameters discussed to study their effects on cancer and total immune cells. Perturbing $\lambda_{VE_{N}}$ shows a time-sensitive effect, showing the early intervention significance in managing cancer progression. The short-lived impact on total immune cells in mouse 1 and the longer-lasting effect in mouse 3 highlight the nuanced responses in different scenarios. This observation aligns with existing literature cautioning against late-stage VEGF therapy for breast cancer, emphasizing timely intervention to yield positive outcomes [128]. Perturbing $\lambda_{OxE_{N}}$ and $Ox_{crit}^{\left(2\right)}$ has a less significant but sustainable effect on total immune cells, especially in mice 1 and 3 over a longer period. While directly controlling hypoxia or oxygen delivery may be challenging, the lasting impact suggests promising therapeutic avenues, as seen in studies targeting the HIF pathways for treatment [129–131].

Our results connect with several clinical trials that have demonstrated the VEGF inhibitors efficacy, such as Bevacizumab, in breast cancer therapy [135]. These inhibitors reduce tumour vasculature, disrupt angiogenesis and indirectly influence oxygenation levels. Importantly, this approach can potentially enhance immuno-therapies efficacy by alleviating hypoxia-induced immunosuppression [136]. Through the PCSA, we observed that targeting VEGF not only disrupts angiogenesis but also reduces the recruitment of regulatory T-cells. This implies a recently discussed function of VEGF and VEGF inhibitors in having a dual effect [137,138]: impairing blood vessel formation while reducing immunosuppression by prohibiting Regulatory T-cell (Treg) proliferation. As discussed, our model and the literature both confirm that this dual mechanism could be especially effective in early tumour stages, enhancing the efficacy of immunotherapies by increasing anti-tumour T-cell responses. Another effect implied by our model (related to the oxygen compartment extension) is the multifaceted benefit of increased IL-12 production in TME through direct cytokine therapy or by stimulating immune cells that produce IL-12. This could provide a novel dual benefit: reducing angiogenesis by inhibiting endothelial cell proliferation and enhancing anti-tumour immune responses by promoting cytotoxic T-cell activity [139,140]. Hence, we hypothesize that IL-12 therapies could be effective when combined with other treatments by weakening tumour vasculature, as they simultaneously attack the tumour’s immune and vascular support systems in breast cancer. These also highlight the importance of personalized treatment strategies that consider the specific angiogenesis and oxygenation dynamics in breast cancer patients. Our work paves the way for the potential *in silico* exploration of the effects of such treatments in future studies.

This study’s findings should be interpreted considering its limitations. One major constraint is the lack of refined time-course data, which, if available, could enhance model precision and prediction reliability. Exploring spatially distributed data and comparing a spatiotemporal version of our model with such data would be extremely interesting. Despite having a sizable system, we acknowledge some key role players in TME are not considered. This constitutes an opportunity for more extensions depending on the particular research needs. Additionally, numerous factors such as the role and type of macrophages [73], as well as the cells’ phenotypic variation under different oxygen conditions [134], are among factors worth further considering in the context of the present network. Lastly, we emphasize the potential metabolic changes occurring in cancer cells, making them nearly immune to hypoxia. This is known as the Warburg effect [141] and is an HIF pathways’ outcome [142]. Despite including the HIF pathways’ effect on the immune system, we did not consider the pathways leading to the Warburg effect for simplicity.

Future studies are needed to overcome the limitations highlighted above, like seeing how the metabolic changes in cancer can affect the system outcome. Moreover, the direct interactions between cancer cells and macrophages can be included in the model by considering the macrophages’ polarization to pro- and anti-tumour subtypes rather than just through the molecular pathways. Furthermore, this model lays the groundwork for developing a TME spatio-temporal PDE model. Such a model can capture interesting phenomena such as VEGFs’ chemotactic signalling to endothelial cells affecting the vessel network formation and location and the oxygen penetration in the tissue influencing the dynamics and shape of the TME.

## Data Availability

Data and relevant code for this research work are stored in GitHub [143] and have been archived within the Zenodo repository [144]. Supplementary material is available online [145].

## References

[1] Kashyap D, Pal D, Sharma R, Garg VK, Goel N, Koundal D, Zaguia A, Koundal S, Belay A. 2022 Global increase in breast cancer incidence: risk factors and preventive measures. Biomed Res. Int. **2022**, 9605439. (10.1155/2022/9605439) 35480139 PMC9038417

[2] Arnold M *et al*. 2022 Current and future burden of breast cancer: global statistics for 2020 and 2040. Breast **66**, 15–23. (10.1016/j.breast.2022.08.010) 36084384 PMC9465273

[3] Siegel RL, Miller KD, Wagle NS, Jemal A. 2023 Cancer statistics. CA Cancer J. Clin. **73**, 17–48. (10.3322/caac.21763) 36633525

[4] Sharma GN, Dave R, Sanadya J, Sharma P, Sharma KK. 2010 Various types and management of breast cancer: an overview. J. Adv. Pharm. Technol. Res. **1**, 109–126. 22247839 PMC3255438

[5] Soysal SD, Tzankov A, Muenst SE. 2015 Role of the tumor microenvironment in breast cancer. Pathobiology **82**, 142–152. (10.1159/000430499) 26330355

[6] Truffi M, Sorrentino L, Corsi F. 2020 Fibroblasts in the tumor microenvironment. In Tumor microenvironment: non-hematopoietic cells. Berlin, Germany: Springer. (10.1007/978-3-030-37184-5_2) 32040852

[7] Elhanani O, Ben-Uri R, Keren L. 2023 Spatial profiling technologies illuminate the tumor microenvironment. Cancer Cell **41**, 404–420. (10.1016/j.ccell.2023.01.010) 36800999

[8] Sharpless NE, Depinho RA. 2006 The mighty mouse: genetically engineered mouse models in cancer drug development. Nat. Rev. Drug Discov. **5**, 741–754. (10.1038/nrd2110) 16915232

[9] Walrath JC, Hawes JJ, Van Dyke T, Reilly KM. 2010 Genetically engineered mouse models in cancer research. Adv. Cancer Res. **106**, 113–164. (10.1016/S0065-230X(10)06004-5) 20399958 PMC3533445

[10] Kersten K, de Visser KE, van Miltenburg MH, Jonkers J. 2017 Genetically engineered mouse models in oncology research and cancer medicine. EMBO Mol. Med. **9**, 137–153. (10.15252/emmm.201606857) 28028012 PMC5286388

[11] Attalla S, Taifour T, Bui T, Muller W. 2021 Insights from transgenic mouse models of pymt-induced breast cancer: recapitulating human breast cancer progression in vivo. Oncogene **40**, 475–491. (10.1038/s41388-020-01560-0) 33235291 PMC7819848

[12] Browne G, Taipaleenmäki H, Bishop NM, Madasu SC, Shaw LM, van Wijnen AJ, Stein JL, Stein GS, Lian JB. 2015 Runx1 is associated with breast cancer progression in MMTV-PyMT transgenic mice and its depletion in vitro inhibits migration and invasion. J. Cell. Physiol. **230**, 2522–2532. (10.1002/jcp.24989) 25802202 PMC4481134

[13] Hallett MA, Teng B, Hasegawa H, Schwab LP, Seagroves TN, Pourmotabbed T. 2013 Anti-matrix metalloproteinase-9 DNAzyme decreases tumor growth in the MMTV-PyMT mouse model of breast cancer. Breast Cancer Res. **15**, R12. (10.1186/bcr3385) 23407024 PMC3672740

[14] Tatarova Z *et al*. 2022 A multiplex implantable microdevice assay identifies synergistic combinations of cancer immunotherapies and conventional drugs. Nat. Biotechnol. **40**, 1823–1833. (10.1038/s41587-022-01379-y) 35788566 PMC9750874

[15] Christenson JL *et al*. 2017 Mmtv-pymt and derived met-1 mouse mammary tumor cells as models for studying the role of the androgen receptor in triple-negative breast cancer progression. Horm. Cancer. **8**, 69–77. (10.1007/s12672-017-0285-6) 28194662 PMC5407486

[16] Almholt K, Juncker-Jensen A, Laerum OD, Danø K, Johnsen M, Lund LR, Rømer J. 2008 Metastasis is strongly reduced by the matrix metalloproteinase inhibitor Galardin in the MMTV-PymT transgenic breast cancer model. Mol. Cancer Ther. **7**, 2758–2767. (10.1158/1535-7163.MCT-08-0251) 18790756

[17] Makhlouf AM, El-Shennawy L, Elkaranshawy HA. 2020 Mathematical modelling for the role of CD4^+^T cells in tumor-immune interactions. Comput. Math. Methods Med. **2020**, 7187602. (10.1155/2020/7187602) 32148558 PMC7049850

[18] Mahlbacher G, Curtis LT, Lowengrub J, Frieboes HB. 2018 Mathematical modeling of tumor-associated macrophage interactions with the cancer microenvironment. J. Immunother. Cancer **6**, 10. (10.1186/s40425-017-0313-7) 29382395 PMC5791333

[19] Mohammad Mirzaei N *et al*. 2021 A mathematical model of breast tumor progression based on immune infiltration. J. Pers. Med. **11**, 1031. (10.3390/jpm11101031) 34683171 PMC8540934

[20] Louzoun Y, Xue C, Lesinski GB, Friedman A. 2014 A mathematical model for pancreatic cancer growth and treatments. J. Theor. Biol. **351**, 74–82. (10.1016/j.jtbi.2014.02.028) 24594371 PMC4011486

[21] Mohammad Mirzaei N, Hao W, Shahriyari L. 2023 Investigating the spatial interaction of immune cells in colon cancer. iScience **26**, 106596. (10.1016/j.isci.2023.106596) 37168560 PMC10165418

[22] Nani F, Freedman HI. 2000 A mathematical model of cancer treatment by immunotherapy. Math. Biosci. **163**, 159–199. (10.1016/S0025-5564(99)00058-9) 10701303

[23] Budithi A, Su S, Kirshtein A, Shahriyari L. 2021 Data driven mathematical model of folfiri treatment for colon cancer. Cancers **13**, 2632. (10.3390/cancers13112632) 34071939 PMC8198096

[24] Le T, Su S, Shahriyari L. 2021 Investigating optimal chemotherapy options for osteosarcoma patients through a mathematical model. Cells **10**, 2009. (10.3390/cells10082009) 34440778 PMC8394778

[25] Brouwer AF, Eisenberg MC, Meza R. 2016 Age effects and temporal trends in HPV-related and HPV-unrelated oral cancer in the United States: a multistage carcinogenesis modeling analysis. PLoS One **11**, e0151098. (10.1371/journal.pone.0151098) 26963717 PMC4786132

[26] Li L, Hu Y, Xu Y, Tang S. 2023 Mathematical modeling the order of driver gene mutations in colorectal cancer. PLoS Comput. Biol. **19**, e1011225. (10.1371/journal.pcbi.1011225) 37368936 PMC10332632

[27] Chaplain MA. 2000 Mathematical modelling of angiogenesis. J. Neurooncol. **50**, 37–51. (10.1023/A:1006446020377) 11245280

[28] Anderson AR, Chaplain MA. 1998 Continuous and discrete mathematical models of tumor-induced angiogenesis. Bull. Math. Biol. **60**, 857–899. (10.1006/bulm.1998.0042) 9739618

[29] Harrington HA, Maier M, Naidoo L, Whitaker N, Kevrekidis PG. 2007 A hybrid model for tumor-induced angiogenesis in the cornea in the presence of inhibitors. Math. Comput. Model. **46**, 513–524. (10.1016/j.mcm.2006.11.034)

[30] Tong S, Yuan F. 2001 Numerical simulations of angiogenesis in the cornea. Microvasc. Res. **61**, 14–27. (10.1006/mvre.2000.2282) 11162192

[31] Peirce SM. 2008 Computational and mathematical modeling of angiogenesis. Microcirculation **15**, 739–751. (10.1080/10739680802220331) 18720228 PMC3125711

[32] Folkman J. 1971 Tumor angiogenesis: therapeutic implications. N. Engl. J. Med. **285**, 1182–1186. (10.1056/NEJM197111182852108) 4938153

[33] Ferrara N, Hillan KJ, Gerber HP, Novotny W. 2004 Discovery and development of bevacizumab, an anti-vegf antibody for treating cancer. Nat. Rev. Drug Discov. **3**, 391–400. (10.1038/nrd1381) 15136787

[34] Mohammad Mirzaei N, Changizi N, Asadpoure A, Su S, Sofia D, Tatarova Z, Zervantonakis IK, Chang YH, Shahriyari L. 2022 Investigating key cell types and molecules dynamics in PyMT mice model of breast cancer through a mathematical model. PLoS Comput. Biol. **18**, e1009953. (10.1371/journal.pcbi.1009953) 35294447 PMC8959189

[35] Mohammad Mirzaei N, Tatarova Z, Hao W, Changizi N, Asadpoure A, Zervantonakis IK, Hu Y, Chang YH, Shahriyari L. 2022 A PDE model of breast tumor progression in MMTV-PyMT mice. J. Pers. Med. **12**, 807. (10.3390/jpm12050807) 35629230 PMC9145520

[36] Cai Y, Nogales-Cadenas R, Zhang Q, Lin JR, Zhang W, O’Brien K, Montagna C, Zhang ZD. 2017 Transcriptomic dynamics of breast cancer progression in the MMTV-PyMT mouse model. BMC Genomics **18**, 185. (10.1186/s12864-017-3563-3) 28212608 PMC5316186

[37] Newman AM *et al*. 2019 Determining cell type abundance and expression from bulk tissues with digital cytometry. Nat. Biotechnol. **37**, 773–782. (10.1038/s41587-019-0114-2) 31061481 PMC6610714

[38] Heng TS, Painter MW, Elpek K, Lukacs-Kornek V, Mauermann N, Turley SJ, Koller D, Kim FS, Wagers AJ. 2008 The immunological genome project: networks of gene expression in immune cells. Nat. Immunol. **9**, 1091–1094. (10.1038/ni1008-1091) 18800157

[39] Tabula Muris Consortium *et al*. 2018 Single-cell transcriptomics of 20 mouse organs creates a tabula muris: the Tabula muris consortium. Nature **562**, 367–372. (10.1038/s41586-018-0590-4) 30283141 PMC6642641

[40] Mirzaei NM, Shahriyari L. 2023 Modeling cancer progression: an integrated workflow extending data-driven kinetic models to bio-mechanical pde models. Phys. Biol **21**, 022001. (10.1088/1478-3975/ad2777) 38330444

[41] Liu Q, Palmgren VAC, Danen EH, Le Dévédec SE. 2022 Acute vs. chronic vs. intermittent hypoxia in breast cancer: a review on its application in in vitro research. Mol. Biol. Rep. **49**, 10961–10973. (10.1007/s11033-022-07802-6) 36057753 PMC9618509

[42] Muz B, de la Puente P, Azab F, Azab AK. 2015 The role of hypoxia in cancer progression, angiogenesis, metastasis, and resistance to therapy. Hypoxia **3**, 83–92. (10.2147/HP.S93413) 27774485 PMC5045092

[43] Vaupel P, Höckel M, Mayer A. 2007 Detection and characterization of tumor hypoxia using po_2_ histography. Antioxid. Redox Signal. **9**, 1221–1235. (10.1089/ars.2007.1628) 17536958

[44] Ebbesen P, Eckardt KU, Ciampor F, Pettersen EO. 2004 Linking measured intercellular oxygen concentration to human cell functions. Acta Oncol. **43**, 598–600. (10.1080/02841860410020220) 15370621

[45] Noman MZ, Hasmim M, Messai Y, Terry S, Kieda C, Janji B, Chouaib S. 2015 Hypoxia: a key player in antitumor immune response. a review in the theme: cellular responses to hypoxia. Am. J. Physiol. Cell Physiol. **309**, C569–79. (10.1152/ajpcell.00207.2015) 26310815 PMC4628936

[46] Liu J *et al*. 2019 CCR7 chemokine receptor-inducible lnc-Dpf3 restrains dendritic cell migration by inhibiting HIF-1α-mediated glycolysis. Immunity **50**, 600–615.(10.1016/j.immuni.2019.01.021) 30824325

[47] Filippi I, Morena E, Aldinucci C, Carraro F, Sozzani S, Naldini A. 2014 Short-term hypoxia enhances the migratory capability of dendritic cell through HIF-1α and PI3K/Akt pathway. J. Cell. Physiol. **229**, 2067–2076. (10.1002/jcp.24666) 24818793

[48] Chen G, Wu K, Li H, Xia D, He T. 2022 Role of hypoxia in the tumor microenvironment and targeted therapy. Front. Oncol. **12**, 961637. (10.3389/fonc.2022.961637) 36212414 PMC9545774

[49] Clambey ET *et al*. 2012 Hypoxia-inducible factor-1 alpha–dependent induction of FoxP3 drives regulatory T-cell abundance and function during inflammatory hypoxia of the mucosa. Proc. Natl Acad. Sci. USA **109**. (10.1073/pnas.1202366109) PMC3478644 22988108

[50] Emami Nejad A *et al*. 2021 The role of hypoxia in the tumor microenvironment and development of cancer stem cell: a novel approach to developing treatment. Cancer Cell Int. **21**. (10.1186/s12935-020-01719-5) PMC7816485 33472628

[51] Gropper Y, Feferman T, Shalit T, Salame TM, Porat Z, Shakhar G. 2017 Culturing CTLs under hypoxic conditions enhances their cytolysis and improves their anti-tumor function. Cell Rep. **20**, 2547–2555. (10.1016/j.celrep.2017.08.071) 28903036

[52] Colegio OR *et al*. 2014 Functional polarization of tumour-associated macrophages by tumour-derived lactic acid. Nature **513**, 559–563. (10.1038/nature13490) 25043024 PMC4301845

[53] Dong H, Zhang L, Liu S. 2022 Targeting hmgb1: an available therapeutic strategy for breast cancer therapy. Int. J. Biol. Sci. **18**, 3421–3434. (10.7150/ijbs.73504) 35637945 PMC9134916

[54] Zhang Y, Yao Y ming, Huang L feng, Dong N, Yu Y, Sheng Z yong. 2011 The potential effect and mechanism of high-mobility group box 1 protein on regulatory T cell-mediated immunosuppression. J. Interferon Cytokine Res. **31**, 249–257. (10.1089/jir.2010.0019) 21087077

[55] Steinman RM, Banchereau J. 2007 Taking dendritic cells into medicine. Nature **449**, 419–426. (10.1038/nature06175) 17898760

[56] Hansen M, Andersen MH. 2017 The role of dendritic cells in cancer. Semin. Immunopathol. **39**, 307–316. (10.1007/s00281-016-0592-y) 27638181

[57] Knutson KL, Disis ML. 2004 IL-12 enhances the generation of tumour antigen-specific Th1 CD4 T cells during ex vivo expansion. Clin. Exp. Immunol. **135**, 322–329. (10.1111/j.1365-2249.2004.02360.x) 14738463 PMC1808930

[58] Cirella A *et al*. 2022 Novel strategies exploiting interleukin-12 in cancer immunotherapy. Pharmacol. Ther. **239**, 108189. (10.1016/j.pharmthera.2022.108189) 35430292

[59] Beyer M, Schultze JL. 2006 Regulatory T cells in cancer. Blood **108**, 804–811. (10.1182/blood-2006-02-002774) 16861339

[60] Whiteside TL. 2012 What are regulatory T cells (Treg) regulating in cancer and why? Semin. Cancer Biol. **22**, 327–334. (10.1016/j.semcancer.2012.03.004) 22465232 PMC3385925

[61] Salkeni MA, Naing A. 2023 Interleukin-10 in cancer immunotherapy: from bench to bedside. Trends Cancer **9**, 716–725. (10.1016/j.trecan.2023.05.003) 37321942 PMC10524969

[62] Sheikhpour E, Noorbakhsh P, Foroughi E, Farahnak S, Nasiri R, Neamatzadeh H. 2018 A survey on the role of interleukin-10 in breast cancer: a narrative. Rep. Biochem. Mol. Biol. **7**, 30–37. 30324115 PMC6175593

[63] Fuertes MB, Kacha AK, Kline J, Woo SR, Kranz DM, Murphy KM, Gajewski TF. 2011 Host type I IFN signals are required for antitumor CD8+ T cell responses through CD8{alpha}+ dendritic cells. J. Exp. Med. **208**, 2005–2016. (10.1084/jem.20101159) 21930765 PMC3182064

[64] Lee YS, Radford KJ. 2019 The role of dendritic cells in cancer. Int. Rev. Cell Mol. Biol. **348**, 123–178. (10.1016/bs.ircmb.2019.07.006) 31810552

[65] Mirlekar B, Pylayeva-Gupta Y. 2021 IL-12 family cytokines in cancer and immunotherapy. Cancers (Basel) **13**, 167. (10.3390/cancers13020167) 33418929 PMC7825035

[66] Watanabe MAE, Oda JMM, Amarante MK, Cesar Voltarelli J. 2010 Regulatory T cells and breast cancer: implications for immunopathogenesis. Cancer Metastasis Rev. **29**, 569–579. (10.1007/s10555-010-9247-y) 20830504

[67] Sato T, Terai M, Tamura Y, Alexeev V, Mastrangelo MJ, Selvan SR. 2011 Interleukin 10 in the tumor microenvironment: a target for anticancer immunotherapy. Immunol. Res. **51**, 170–182. (10.1007/s12026-011-8262-6) 22139852

[68] Palucka K, Coussens LM, O’Shaughnessy J. 2013 Dendritic cells, inflammation and breast cancer. Cancer J. **19**, 511–516. (10.1097/PPO.0000000000000007) 24270350 PMC3886565

[69] Tran Janco JM, Lamichhane P, Karyampudi L, Knutson KL. 2015 Tumor-infiltrating dendritic cells in cancer pathogenesis. J. Immunol. **194**, 2985–2991. (10.4049/jimmunol.1403134) 25795789 PMC4369768

[70] Marciscano AE, Anandasabapathy N. 2021 The role of dendritic cells in cancer and anti-tumor immunity. Semin. Immunol. **52**, 101481. (10.1016/j.smim.2021.101481) 34023170 PMC8545750

[71] Tripathi A, Shrinet K, Kumar A. 2019 HMGB1 protein as a novel target for cancer. Toxicol. Rep. **6**, 253–261. (10.1016/j.toxrep.2019.03.002) 30911468 PMC6416660

[72] Campana L, Bosurgi L, Rovere-Querini P. 2008 HMGB1: a two-headed signal regulating tumor progression and immunity. Curr. Opin. Immunol. **20**, 518–523. (10.1016/j.coi.2008.04.012) 18599281

[73] Bull JA, Byrne HM. 2023 Quantification of spatial and phenotypic heterogeneity in an agent-based model of tumour-macrophage interactions. PLOS Comput. Biol. **19**, e1010994. (10.1371/journal.pcbi.1010994) 36972297 PMC10079237

[74] Hao NB, Lü MH, Fan YH, Cao YL, Zhang ZR, Yang SM. 2012 Macrophages in tumor microenvironments and the progression of tumors. Clin. Dev. Immunol. **2012**, 1–11. (10.1155/2012/948098) PMC3385963 22778768

[75] Mirlekar B. 2022 Tumor promoting roles of IL-10, TGF-β, IL-4, and IL-35: Its implications in cancer immunotherapy. SAGE Open Med. **10**, 20503121211069012. (10.1177/20503121211069012) 35096390 PMC8793114

[76] Müller AK *et al*. 2022 Mouse modeling dissecting macrophage-breast cancer communication uncovered roles of PYK2 in macrophage recruitment and breast tumorigenesis. Adv. Sci. **9**, e2105696. (10.1002/advs.202105696) PMC8948556 35092356

[77] Steding CE, Wu S tse, Zhang Y, Jeng MH, Elzey BD, Kao C. 2011 The role of interleukin-12 on modulating myeloid-derived suppressor cells, increasing overall survival and reducing metastasis. Immunology **133**, 221–238. (10.1111/j.1365-2567.2011.03429.x) 21453419 PMC3088984

[78] Aras S, Zaidi MR. 2017 Tameless traitors: macrophages in cancer progression and metastasis. Br. J. Cancer **117**, 1583–1591. (10.1038/bjc.2017.356) 29065107 PMC5729447

[79] Chen Y, Song Y, Du W, Gong L, Chang H, Zou Z. 2019 Tumor-associated macrophages: an accomplice in solid tumor progression. J. Biomed. Sci. **26**, 78. (10.1186/s12929-019-0568-z) 31629410 PMC6800990

[80] Di GH, Liu Y, Lu Y, Liu J, Wu C, Duan HF. 2014 Il-6 secreted from senescent mesenchymal stem cells promotes proliferation and migration of breast cancer cells. PLoS One **9**, e113572. (10.1371/journal.pone.0113572) 25419563 PMC4242635

[81] Guo Y, Xu F, Lu T, Duan Z, Zhang Z. 2012 Interleukin-6 signaling pathway in targeted therapy for cancer. Cancer Treat. Rev. **38**, 904–910. (10.1016/j.ctrv.2012.04.007) 22651903

[82] Wang S, Su X, Xu M, Xiao X, Li X, Li H, Keating A, Zhao RC. 2019 Exosomes secreted by mesenchymal stromal/stem cell-derived adipocytes promote breast cancer cell growth via activation of Hippo signaling pathway. Stem Cell Res. Ther. **10**. (10.1186/s13287-019-1220-2) PMC6458638 30971292

[83] Gao Y *et al*. 2020 Adipocytes promote breast tumorigenesis through TAZ-dependent secretion of Resistin. Proc. Natl Acad. Sci. USA **117**, 33295–33304. (10.1073/pnas.2005950117) 33318171 PMC7776784

[84] Durgeau A, Virk Y, Corgnac S, Mami-Chouaib F. 2018 Recent advances in targeting cd8 t-cell immunity for more effective cancer immunotherapy. Front. Immunol. **9**, 14. (10.3389/fimmu.2018.00014) 29403496 PMC5786548

[85] Raskov H, Orhan A, Christensen JP, Gögenur I. 2021 Cytotoxic CD8+ T cells in cancer and cancer immunotherapy. Br. J. Cancer **124**, 359–367. (10.1038/s41416-020-01048-4) 32929195 PMC7853123

[86] West J, Newton PK. 2019 Cellular interactions constrain tumor growth. Proc. Natl Acad. Sci. USA **116**, 1918–1923. (10.1073/pnas.1804150116) 30674661 PMC6369808

[87] Rozhok A, DeGregori J. 2019 A generalized theory of age-dependent carcinogenesis. eLife **8**, e39950. (10.7554/eLife.39950) 31034356 PMC6488293

[88] Liu Z gang, Jiao D. 2020 Necroptosis, tumor necrosis and tumorigenesis. Cell Stress **4**, 1–8. (10.15698/cst2020.01.208) PMC6946014 31922095

[89] Kang R, Zhang Q, Zeh HJ III, Lotze MT, Tang D. 2013 HMGB1 in cancer: good, bad, or both? Clin. Cancer Res. **19**, 4046–4057. (10.1158/1078-0432.CCR-13-0495) 23723299 PMC3732559

[90] Ma X, Yan W, Zheng H, Du Q, Zhang L, Ban Y, Li N, Wei F. 2015 Regulation of IL-10 and IL-12 production and function in macrophages and dendritic cells. F1000Res **4**. (10.12688/f1000research.7010.1) PMC4754024 26918147

[91] Chen J, Wei Y, Yang W, Huang Q, Chen Y, Zeng K, Chen J. 2022 IL-6: the link between inflammation, immunity and breast cancer. Front. Oncol. **12**, 903800. (10.3389/fonc.2022.903800) 35924148 PMC9341216

[92] Luan D, Dadpey B, Zaid J, Bridge-Comer PE, DeLuca JH, Xia W, Castle J, Reilly SM. 2023 Adipocyte-secreted IL-6 sensitizes macrophages to IL-4 signaling. Diabetes **72**, 367–374. (10.2337/db22-0444) 36449000 PMC9935493

[93] Mühleder S, Fernández-Chacón M, Garcia-Gonzalez I, Benedito R. 2021 Endothelial sprouting, proliferation, or senescence: tipping the balance from physiology to pathology. Cell. Mol. Life Sci. **78**, 1329–1354. (10.1007/s00018-020-03664-y) 33078209 PMC7904752

[94] Vona-Davis L, Rose DP. 2009 Angiogenesis, adipokines and breast cancer. Cytokine Growth Factor Rev. **20**, 193–201. (10.1016/j.cytogfr.2009.05.007) 19520599

[95] Cao Y. 2007 Angiogenesis modulates adipogenesis and obesity. J. Clin. Invest. **117**, 2362–2368. (10.1172/JCI32239) 17786229 PMC1963348

[96] Nyberg P, Xie L, Kalluri R. 2005 Endogenous inhibitors of angiogenesis. Cancer Res. **65**, 3967–3979. (10.1158/0008-5472.CAN-04-2427) 15899784

[97] Fox SB, Generali DG, Harris AL. 2007 Breast tumour angiogenesis. Breast Cancer Res. **9**. (10.1186/bcr1796) PMC2246178 18190723

[98] Ferrara N. 2002 Vegf and the quest for tumour angiogenesis factors. Nat. Rev. Cancer **2**, 795–803. (10.1038/nrc909) 12360282

[99] Anders S, Huber W. 2010 Differential expression analysis for sequence count data. Nat. Prec. 1. (10.1038/npre.2010.4282.2) PMC3218662 20979621

[100] Le T, Aronow RA, Kirshtein A, Shahriyari L. 2021 A review of digital cytometry methods: estimating the relative abundance of cell types in a bulk of cells. Brief. Bioinform. **22**, 07. (10.1093/bib/bbaa219) PMC8293826 33003193

[101] Aronow RA, Akbarinejad S, Le T, Su S, Shahriyari L. 2022 TumorDecon: a digital cytometry software. SoftwareX **18**, 101072. (10.1016/j.softx.2022.101072) 35782394 PMC9248989

[102] Sun L, Han X, Egeblad M. 2022 Isolation of mouse mammary carcinoma-derived macrophages and cancer cells for co-culture assays. STAR Protoc. **3**, 101833. (10.1016/j.xpro.2022.101833) 36386879 PMC9664409

[103] Sun L *et al*. 2021 Activating a collaborative innate-adaptive immune response to control metastasis. Cancer Cell **39**, 1361–1374. (10.1016/j.ccell.2021.08.005) 34478639 PMC8981964

[104] Tasnim H, Fricke GM, Byrum JR, Sotiris JO, Cannon JL, Moses ME. 2018 Quantitative measurement of naïve T cell association with dendritic cells, FRCs, and blood vessels in lymph nodes. Front. Immunol. **9**, 1571. (10.3389/fimmu.2018.01571) 30093900 PMC6070610

[105] Chitu V, Yeung YG, Yu W, Nandi S, Stanley ER. 2011 Measurement of macrophage growth and differentiation. Curr. Protoc. Immunol. (10.1002/0471142735.im1420s92) PMC4184440 21400680

[106] Kornmann LM, Zernecke A, Curfs DMJ, Janssen BJA, Weber C, de Winther MPJ, Reneman RS, Hoeks APG, Reesink KD. 2015 Echogenic perfluorohexane-loaded macrophages adhere in vivo to activated vascular endothelium in mice, an explorative study. Cardiovasc. Ultrasound **13**, 1. (10.1186/1476-7120-13-1) 25567641 PMC4293794

[107] O’Connell KE *et al*. 2015 Practical murine hematopathology: a comparative review and implications for research. Comp. Med. **65**, 96–113. 25926395 PMC4408895

[108] Pillarisetti A, Ladjal H, Ferreira A, Keefer C, Desai JP. 2009 Mechanical characterization of mouse embryonic stem cells. In In 2009 Annual International Conference of the IEEE Engineering in Medicine and Biology Society, pp. 1176–1179. IEEE. (10.1109/IEMBS.2009.5333954) 19964500

[109] Jiang T *et al*. 2023 Matrix mechanics regulate the polarization state of bone marrow-derived neutrophils through the JAK1/STAT3 signaling pathway. Acta Biomater. **168**, 159–173. (10.1016/j.actbio.2023.07.012) 37467837

[110] Chen W, Wei W, Yu L, Zhang X, Huang F, Zheng Q, Wang L, Cai C. 2021 Baicalin promotes mammary gland development via steroid-like activities. Front. Cell Dev. Biol. **9**, 682469. (10.3389/fcell.2021.682469) 34295892 PMC8290356

[111] Laget S *et al*. 2017 Technical insights into highly sensitive isolation and molecular characterization of fixed and live circulating tumor cells for early detection of tumor invasion. PLoS One **12**, e0169427. (10.1371/journal.pone.0169427) 28060956 PMC5218415

[112] Hoogduijn MJ, van den Beukel JC, Wiersma LCM, Ijzer J. 2013 Morphology and size of stem cells from mouse and whale: observational study. BMJ **347**, f6833. (10.1136/bmj.f6833) 24336001 PMC3898169

[113] Turgay Y *et al*. 2017 The molecular architecture of lamins in somatic cells. Nature **543**, 261–264. (10.1038/nature21382) 28241138 PMC5616216

[114] Hagberg CE *et al*. 2018 Flow cytometry of mouse and human adipocytes for the analysis of browning and cellular heterogeneity. Cell Rep. **24**, 2746–2756.(10.1016/j.celrep.2018.08.006) 30184507 PMC6137819

[115] Yamamoto A, Huang Y, Krajina BA, McBirney M, Doak AE, Qu S, Wang CL, Haffner MC, Cheung KJ. 2023 Metastasis from the tumor interior and necrotic core formation are regulated by breast cancer-derived angiopoietin-like 7. Proc. Natl Acad. Sci. USA **120**, e2214888120. (10.1073/pnas.2214888120) 36853945 PMC10013750

[116] El-Mihoub TA, Hopgood AA, Nolle L, Battersby A. 2006 Hybrid genetic algorithms: a review. Eng. Lett. **13**, 124–137.

[117] Holland JH. 1992 Adaptation in natural and artificial systems: an introductory analysis with applications to biology, control, and artificial intelligence. Cambridge, MA: MIT press.

[118] Villaverde AF, Tsiantis N, Banga JR. 2019 Full observability and estimation of unknown inputs, states and parameters of nonlinear biological models. J. R. Soc. Interface **16**, 20190043. (10.1098/rsif.2019.0043) 31266417 PMC6685009

[119] Villaverde AF, Barreiro A, Papachristodoulou A. 2016 Structural identifiability of dynamic systems biology models. PLoS Comput. Biol. **12**, e1005153. (10.1371/journal.pcbi.1005153) 27792726 PMC5085250

[120] Villaverde AF, Evans ND, Chappell MJ, Banga JR. 2018 Input-dependent structural identifiability of nonlinear systems. IEEE Control Syst. Lett. **3**, 272–277. (10.1109/LCSYS.2018.2868608)

[121] Massonis G, Villaverde AF. 2020 Finding and breaking lie symmetries: implications for structural identifiability and observability in biological modelling. Symmetry **12**, 469. (10.3390/sym12030469)

[122] Massonis G, Banga JR, Villaverde AF. 2023 AutoRepar: a method to obtain identifiable and observable reparameterizations of dynamic models with mechanistic insights. Intl. J. Robust Nonlinear **33**, 5039–5057. (10.1002/rnc.5887)

[123] Wieland FG, Hauber AL, Rosenblatt M, Tönsing C, Timmer J. 2021 On structural and practical identifiability. Curr. Opin. Syst. Biol. **25**, 60–69. (10.1016/j.coisb.2021.03.005)

[124] Raue A, Kreutz C, Maiwald T, Bachmann J, Schilling M, Klingmüller U, Timmer J. 2009 Structural and practical identifiability analysis of partially observed dynamical models by exploiting the profile likelihood. Bioinformatics **25**, 1923–1929. (10.1093/bioinformatics/btp358) 19505944

[125] Domijan M, Brown PE, Shulgin BV, Rand DA. 2016 Pettsy: a computational tool for perturbation analysis of complex systems biology models. BMC Bioinformatics **17**, 124. (10.1186/s12859-016-0972-2) 26964749 PMC4785672

[126] Soguel L, Durocher F, Tchernof A, Diorio C. 2017 Adiposity, breast density, and breast cancer risk: epidemiological and biological considerations. Eur. J. Cancer Prev. **26**, 511–520. (10.1097/CEJ.0000000000000310) 27571214 PMC5627530

[127] DeBerardinis RJ, Chandel NS. 2020 We need to talk about the Warburg effect. Nat. Metab. **2**, 127–129. (10.1038/s42255-020-0172-2) 32694689

[128] Bergers G, Hanahan D. 2008 Modes of resistance to anti-angiogenic therapy. Nat. Rev. Cancer **8**, 592–603. (10.1038/nrc2442) 18650835 PMC2874834

[129] Milani M, Harris AL. 2008 Targeting tumour hypoxia in breast cancer. Eur. J. Cancer **44**, 2766–2773. (10.1016/j.ejca.2008.09.025) 18990559

[130] Onnis B, Rapisarda A, Melillo G. 2009 Development of hif-1 inhibitors for cancer therapy. J. Cell. Mol. Med. **13**, 2780–2786. (10.1111/j.1582-4934.2009.00876.x) 19674190 PMC2832082

[131] Semenza GL. 2003 Targeting hif-1 for cancer therapy. Nat. Rev. Cancer **3**, 721–732. (10.1038/nrc1187) 13130303

[132] Nieman KM, Romero IL, Van Houten B, Lengyel E. 2013 Adipose tissue and adipocytes support tumorigenesis and metastasis. Biochim. Biophys. Acta **1831**, 1533–1541. (10.1016/j.bbalip.2013.02.010) 23500888 PMC3742583

[133] Trayhurn P. 2013 Hypoxia and adipose tissue function and dysfunction in obesity. Physiol. Rev. **93**, 1–21. (10.1152/physrev.00017.2012) 23303904

[134] Celora GL, Byrne HM, Zois CE, Kevrekidis PG. 2021 Phenotypic variation modulates the growth dynamics and response to radiotherapy of solid tumours under normoxia and hypoxia. J. Theor. Biol. **527**, 110792. (10.1016/j.jtbi.2021.110792) 34087269

[135] Miller K, Wang M, Gralow J, Dickler M, Cobleigh M, Perez EA, Shenkier T, Cella D, Davidson NE. 2007 Paclitaxel plus bevacizumab versus paclitaxel alone for metastatic breast cancer. N. Engl. J. Med. **357**, 2666–2676. (10.1056/NEJMoa072113) 18160686

[136] Jain RK. 2005 Normalization of tumor vasculature: an emerging concept in antiangiogenic therapy. Science **307**, 58–62. (10.1126/science.1104819) 15637262

[137] Chen W, Shen L, Jiang J, Zhang L, Zhang Z, Pan J, Ni C, Chen Z. 2021 Antiangiogenic therapy reverses the immunosuppressive breast cancer microenvironment. Biomark. Res. **9**, 59. (10.1186/s40364-021-00312-w) 34294146 PMC8296533

[138] Zhang Y, Brekken RA. 2022 Direct and indirect regulation of the tumor immune microenvironment by VEGF. J. Leukoc. Biol. **111**, 1269–1286. (10.1002/JLB.5RU0222-082R) 35466428 PMC9148445

[139] Minnar CM, Lui G, Gulley JL, Schlom J, Gameiro SR. 2023 Preclinical and clinical studies of a tumor targeting IL-12 immunocytokine. Front. Oncol. **13**, 1321318. (10.3389/fonc.2023.1321318) 38260854 PMC10802843

[140] Xu Y, Sun X, Tong Y. 2024 Interleukin-12 in multimodal tumor therapies for induction of anti-tumor immunity. Discov. Oncol. **15**, 170. (10.1007/s12672-024-01011-2) 38753073 PMC11098992

[141] Liberti MV, Locasale JW. 2016 The warburg effect: how does it benefit cancer cells? Trends Biochem. Sci. **41**, 211–218. (10.1016/j.tibs.2015.12.001) 26778478 PMC4783224

[142] Courtnay R, Ngo DC, Malik N, Ververis K, Tortorella SM, Karagiannis TC. 2015 Cancer metabolism and the warburg effect: the role of hif-1 and pi3k. Mol. Biol. Rep. **42**, 841–851. (10.1007/s11033-015-3858-x) 25689954

[143] nmirzaei. 2024 Oxygen-angiogenesis-cancer-and-immune-interplay-in-breast-TME-A-computational-investigation. GitHub. See https://github.com/nmirzaei/Oxygen-Angiogenesis-Cancer-and-Immune-Interplay-in-Breast-TME-A Computational-Investigation. 10.1098/rsos.240718 PMC11631512 39665095

[144] Mirzaei NM. 2024 nmirzaei/Oxygen-angiogenesis-cancer-and-immune-interplay-in-breast-TME-A-computational-investigation. Zenodo. (10.5281/zenodo.13904703) PMC11631512 39665095

[145] Mohammad Mirzaei N, Kevrekidis PG, Shahriyari L. 2024 Supplementary material from: Oxygen, Angiogenesis, Cancer and Immune Interplay in Breast Tumor Micro-Environment: A Computational Investigation. FigShare (10.6084/m9.figshare.c.7574999) PMC11631512 39665095

